# Genome analysis and machine learning-based feature selection strategy reveal potential drug-resistance determinants in *Nakaseomyces glabratus*

**DOI:** 10.1080/22221751.2025.2595789

**Published:** 2025-12-13

**Authors:** Qiqi Wang, Runhong Chen, Xin Cao, Hao Zhang, Jielin Yang, Xinlong Wang, Yadong Liu, Xinyu Tan, Tianyu Liang, Ruoyu Li, Zhe Wan, Yejun Wang, Wei Liu

**Affiliations:** a Department of Dermatology and Venerology, Peking University First Hospital, National Clinical Research Center for Skin and Immune Diseases, Research Center for Medical Mycology, Peking University, Beijing Key Laboratory of Molecular Diagnosis on Dermatoses, Beijing, People’s Republic of China; b Youth Innovation Team of Medical Bioinformatics, Shenzhen University Health Science Center, Shenzhen, People’s Republic of China; c Department of Dermatology, Jiangxi Provincial Children's Hospital, The Affiliated Children's Hospital of Nanchang Medical College, Nanchang, People’s Republic of China; d State Key Laboratory of Microbial Resources, Institute of Microbiology, Chinese Academy of Sciences, University of Chinese Academy of Sciences, Beijing, People’s Republic of China

**Keywords:** *Nakaseomyces glabratus*, antifungal resistance, genome analysis, machine learning, genomic signatures, resistance determinants

## Abstract

Invasive candidiasis caused by *Nakaseomyces glabratus* is of great concern due to high morbidity and mortality, especially antifungal resistance. To identify genomic signatures, which significantly link to drug-resistance, is of great significance in combating this lethal disease. In this study, we performed whole genome analysis on 109 clinical strains of *N. glabratus* which had been isolated from multi-centres in China. By using genome-wide association studies (GWAS), genomic signatures, including several *PDR1* mutations and genes encoding GLEYA-containing proteins, were identified to be significantly linked to drug-resistance. With the strategy of feature-selection combining machine-learning (ML), more relevant genomic signatures and potential resistance determinants were identified, including Y682C and I380L mutations in *PDR1* which were further confirmed to confer triazole-resistance by gene editing technology. We believe that the ML-based feature selection (MLFS) strategy, which is based on a comprehensive understanding of genomic characteristics as described in this study, shows excellent capacity to predict resistance and potential resistance determinants in *N. glabratus*.

## Introduction

*Nakaseomyces glabratus*, previously named *Candida glabrata*, is a globally distributed commensal pathogenic yeast causing invasive candidiasis (IC), with mortality as high as 20%−50% [1]. Although echinocandins are the first-line therapy for IC, triazoles are recommended as initial therapy in meningitis and urinary tract infections [2]. In recent years, resistance to triazoles and echinocandins in *N. glabratus* has increased and resulted in the high rate of mortality in IC caused by *N. glabratus* [3]. Considering its public health importance and especially antifungal resistance, the World Health Organization fungal priority pathogens list ranked *N. glabratus* as a high priority group [4].

Phylogenetically, *N. glabratus* is closely related to the non-pathogenic yeast *Saccharomyces cerevisiae* instead of *Candida* pathogens [5]. And *N. glabratus* is remarkably different from other *Candida* pathogens such as *C. albicans* [5] in the morphology, pathogenesis, stress adaptation, antifungal resistance and evolutionary routes. More notably, it is the mutations in the transcriptional factor *PDR1* that result in triazole-resistance in *N. glabratus*, while the mutations in the triazole target gene *ERG11* [6] mainly confers triazole-resistance in other *Candida* pathogens. Therefore, it is of great significance in elaborating the genomic characteristics associating with pathogenic characters and antifungal susceptibility in *N. glabratus*. Although several reports on the *N. glabratus* genome have been published previously [7, 8], more studies describing genome characters are still needed, especially those with high-quality genome assemblies, population genomic analysis and comprehensive genome-wide association analysis.

Multiple strategies have been used to link genomic information with drug resistance. A common method is genome-wide association studies (GWAS), which test statistical associations between genetic variants in whole genomes and resistant phenotypes to discover potential resistance determinants. The more updated strategies are machine learning (ML) models, which provide accurate predictions of resistance when trained on sufficient data [9]. ML models have been developed to predict drug resistance and putative resistance determinants in bacteria including *Mycobacterium tuberculosis*, *Salmonella* spp., and *Escherichia coli* [10]. However, ML models have rarely been used to predict antifungal-resistance in pathogenic fungi, except for predicting triazole- and echinocandin-resistance in *C. auris* [11]. To our knowledge, it could also be one of the earliest attempts to apply the ML strategy in disclosing new mechanisms underlying antifungal resistance.

In the present study, we performed whole-genome sequencing (WGS) and pan-genome analysis on 109 clinical isolates of *N. glabratus*, revealing high plasticity of the accessory genome composed of horizontal gene transfers (HGT). We also performed GWAS and identified genomic signatures associated with drug resistance. In addition, we established a machine learning-based feature selection (MLFS) strategy to predict triazole resistance and potential-resistance determinants, which were further confirmed by using CRISPR-based gene editing method.

## Materials and methods

### Isolation and species identification of *N. glabratus* strains

Clinical isolates of *N. glabratus* were obtained from CARST-fungi study [12] or preserved at Research Center for Medical Mycology, Peking University, Beijing, China. Isolates were routinely grown on YPD medium (1% yeast extract, 2% peptone, and 2% glucose. 2% Bacto agar was added for solid medium [all from BD Biosciences]). Species identification was performed by sequence-based methods for the internal transcribed spacer (ITS) region and 28S ribosomal subunit (D1/D2) [12].

### Antifungal susceptibility testing

Antifungal susceptibility testing was performed according to the Clinical and Laboratory Standards Institute (CLSI) M27-A4 microbroth dilution method. The tested drugs included fluconazole (FLC), itraconazole (ITC), voriconazole (VRC), posaconazole (POS), caspofungin (CAS), anidulafungin (ANF), micafungin (MCF), and amphotericin B (AMB, all from Harveybio Gene Technology Co. Ltd., Beijing, China). The interpretation of susceptibility was determined by the clinical breakpoints (CBPs) for FLC, CAS, ANF, MCF according to CLSI M60 document. In the absence of CBPs, isolates were defined as wild-type (WT) or a non-WT (NWT) to ITC, VRC, POS, AMB according to the epidemiological cutoff values (ECVs) determined by CLSI M59 document. In the context, to observe the general genetic features associated with drug resistance, we also classify the isolates into anti-microbial resistant (AMR) and sensitive (SEN), which represent resistance to at least one type of the above drugs and sensitivity to all the above drugs, respectively. Similarly, the isolates were also classified into MDR and non-MDR, representing resistance to more than one type of the above drugs and the others, respectively.

### Genome sequencing, assembly, and annotation

The genome sequencing and analytic procedure referred to Supplemental Figure S1. Each clinical isolate was inoculated on a YPD agar plate, and a single colony from primary culture plate was subcultured in YPD medium to ensure purity. Genomic DNA was extracted using DNeasy Plant Mini Kit (Qiagen, Hilden, Germany). DNA concentration and quality were measured using NanoDrop 2000 (ThermoFisher) and agarose gel electrophoresis. Approximately 1 μg genomic DNA from each isolate was used to construct the library with MGIEasy Universal DNA Library Prep Set (Cat # 1000005250, BGI, China). The libraries were sequenced with a BGISEQ-2000 platform (BGI, China). Raw data were trimmed using Trim-Galore (v0.6.10) with parameters of “–quality 20 –stringency 10 -length 75” [13]. Adapters were automatically searched for and removed. Sequences with quality scores above 20 were retained. SPAdes (v3.15.4) [14] was used to make de novo assembly of the clean data into contigs, with the ones larger than 1 kilobase retained. Genome completeness and contamination were assessed using BUSCO v5.4.2 with genome mode based on the data set fungi_odb10 (2021-06-28) [15]. The contigs of each isolate were also mapped to the reference genome of *N. glabratus* CBS138 (NC_005967.2) with BLASTn to evaluate the coverage. The genes of assembled genomes were predicted using GeneMark ES [16] and YGAP [17]. The function of derived peptide sequences was annotated by mapping to both the “fungi” and default protein and domain database with eggNOG-mapper v.4.5.1 [18], with default database annotation for the un-annotated proteins by the “fungi” database.

### Pan-genome analysis

The tool, Get_homologues [19], was used to analyse the pan-genome of the 109 *N. glabratus* isolates. Both diamond and blast were tested, but the latter was used eventually since the potentially higher accuracy though with a tradeoff of computational cost. The homology threshold for family clustering was set as 0.7. A new blastp-based parallelized tool, BactPG2.0, was also applied with default parameters (the product of mutual protein coverage and identity being not smaller than 0.7) to retrieve the pan gene set from the genome of *N. glabratus* (http://61.160.194.165:3080/ESG/tools/). Comparison between the results from Get_homologues and BactPG2.0 showed a large consistency with each other (96.4%), with only a few discordant families. These were manually checked and corrected according to the family member composition, genome adjacency and positioning relationship to other orthologous families. The orphan families, which are only distributed in a single isolate, were removed for further analysis, to further limit the influence caused by possible contaminations. A variety of strains were sampled from the total 109 *N. glabratus* isolates, while the pan- and core-genome families (presented in all the isolates) were analysed repeatedly and plotted against the number of isolates. The total pan gene sets were further divided into core genes, softcore genes, shell genes and cloud genes, defined as the corresponding gene families shared by 100%, > =  95% but  <  100%, > =  5% but  <  95% and  <  5% isolates, respectively. Shell and cloud genes were considered as accessory genes in the study. A representative gene was selected from each pan-genome family and its function annotation information was retrieved. Functional enrichment analysis was performed to groups of gene families, such as the accessory gene families. Fisher exact tests with Bonferroni corrections were adopted for the functional enrichment analysis, using all the genome-derived proteins of CBS138 as the background. The significance was preset as an adjusted *P*-value of being smaller than 0.05.

### Phylogenomic analysis

The single-copy core gene families were further used for phylogenomic analysis. The protein sequences were aligned with clustalw2 for each core gene family. The polymorphic sites were identified, extracted and concatenated from the multiple sequence alignment (MSA) results of all the core gene families, followed by tree building with MEGA 7.0 and a Neighbor-Joining method [20]. Bootstrapping tests were repeated for 1000 rounds to examine the robustness of phylogenetic nodes. An R package ggtree was used to display detailed information about the isolates [21].

### Correlation analysis between antifungal resistance and phylogenetics, isolation place and year

For each antifungal, a general linear model (GLM) was fit, with resistance phenotype as the dependent variable, and phylogenetic cluster, isolation place and isolation year as covariates. The phenotype being resistant or susceptible to an antifungal was denoted as 1 or 0, respectively. The phylogenetic clusters were recognized according to the branch length and encoded as sequential numbers based on the phylogenetic relationship. The beta co-efficient for each covariate was calculated for each model, and the two-tail Student’s t-test was performed to test the significance of the correlation between each covariate and the dependent variable, with Benjamini-Hochberg corrections for multiple tests. The statistical rejection level was preset as a false discovery rate (FDR) being smaller than 0.05. An R package “stats” was used to develop the GLM models.

### Analysis of gene duplications

The pan-genome families with multiple copies of gene members in one or more isolates were retrieved, for which duplications happened. Fragmental duplications involving multiple genes were detected manually by the positional relationship of the examined duplicated genes within the same isolates. Functional enrichment analysis was performed on the families with gene duplications, using the similar strategy described above.

### Association analysis between drug resistance and genome-wide single nucleotide polymorphisms

The genomes of *N. glabratus* isolates were aligned to the reference genome (GCF_000002545.3) with BWA [22]. Samtools V1.13 [23], GATK V4.4 [24] and bcftools V [23] 1.13 were used to convert the file formats of alignment results and retrieve the single nucleotide polymorphism (SNP) results. The low-quality SNP sites were filtered, and association analysis was performed with PLINK V1.90 [25] to test the correlation between the remaining SNPs and drug resistance. Manhattan maps were performed with the qqman package [26]. The significant associations were defined with a *P*-value lower than 1e-05.

### Identification of resistance-related genomic signatures

Signatures were identified in both core-genome and pan-genome from the isolates resistant to each antifungal drug. The composition genes and featured polymorphic amino acids were identified as pan-genome and core-genome signatures, respectively. The frequency of the presence of each pan-genome family in drug-resistant and sensitive strains was compared using EBT [27]. The top 500 genes with significantly different composition were retained, followed by relevance analysis with mRMR [28]. At the core-genome level, the sites with polymorphic amino acid composition were identified by the MSA. For each polymorphic site, the frequency of amino acids with minor composition was compared between drug-resistant and sensitive strains with EBT. The top 500 significant sites were retained for relevance analysis with mRMR. The most relevant n polymorphic sites were selected as the signatures, whereas n was chosen according to the context. The capability of resistance prediction was evaluated by the PCA and T-SNE coordinates of the dimensions with the most relevant information.

### Machine learning models predicting drug-resistance

ML models were trained with core-genome and pan-genome signatures to predict the resistance of *N. glabratus* isolates to azoles. For models based on core-genome signatures, each isolate was represented as a vector of “1” and “0”, with “1” meant the composition of a featured amino acid in the signature site, while “0” meant the otherwise. For pan-genome models, each isolate was also represented as a vector of “1” and “0”, which indicated the presence or absence of the featured gene family, respectively. We also tested other types of features, such as CNV, and developed extended pan-genome (ePG) models, for which the presence and copy number of each featured pan-genome family were represented in 2-bit “0/1”, with “00” meaning absence of the family in the individual strain, “01” meaning presence of only one copy of the family, and “11” meaning presence of multiple copies of the family. The ePG features were selected with a similar strategy mentioned above as for pan-genome features. To confirm the effectiveness of each group of signatures in predicting drug-resistance, permutation analysis was performed, for which the phenotype label of drug resistance was permutated, and the models were trained with the same sets of signatures to predict the newly labelled ones. Various ML algorithms and models were examined and trained, including Logistic Regression (LR), Support Vector Machine (SVM), Random Forest (RF) and Naïve Bayesian (NB). The Python package scikit-learn was used to train the models while Bayesian optimization algorithms were applied to optimize the parameters [29]. The optimized parameters for each pan-genome or core-genome model were provided in Supplemental Dataset S18. A five-fold cross-validation strategy was used to assess the performance of models predicting drug resistance. For each drug-resistance phenotype, the resistant and sensitive isolates were divided into five aliquots, four of which were used as the training dataset and the remaining one aliquot as the test dataset. The performance of models was evaluated on the test datasets. The Receiver Operating Characteristic (ROC) curves were plotted for each model according to its predictions on each testing dataset. The Area Under the Curve (rocAUC) was calculated for each ROC curve. Youden index was maximized to determine the diagnostic cutoff for each model, and the true positive (TP), true negative (TN), false positive (FP) and false negative (FN) predictions could be counted, and Sensitivity, Specificity and Accuracy were calculated:

Sensitivity=TP/(TP+FN),Specificity=TN/(TN+FP),Accuracy=(TP+TN)/(TP+FN+TN+FP).

The measure rocAUC was used as the main item for performance assessment since it is not influenced by the diagnostic cutoff, which could be shifted to balance sensitivity and specificity.

### CRISPR-Cas9-based gene editing

Strains with I380L and Y682C mutations in *PDR1* were constructed in *N. glabratus* reference strain ATCC2001 by CRISPR-based mutagenesis as described previously [30], except for the following modification. Repair DNA templates containing *PDR1* gene fused with *NAT1* gene (downstream) encoding nourseothricin acetyltransferase as a selection marker were generated. Upstream *PDR1* regions containing the mutations were amplified from triazole-resistant clinical *N. glabratus* isolates BMU10123 and BMU06078, which harboured I380L and Y682C mutations in *PDR1*, respectively. Because these fragments also contained the PAM region (short NGG sequence that follows the DNA region targeted for cleavage by the CRISPR system), additional synonymous mutations in the PAM region were performed to bypass recutting by the Cas9. The downstream *PDR1* region (about 0.4 Kb) was amplified from *N. glabratus* ATCC2001, and *NAT1* gene was amplified from a vector pv1326. The oligonucleotides used are listed in Table S1. Transformations were performed as described previously [30]. Immediately following transformation, cells were resuspended in 1 mL ice-cold 1 M sorbitol and were recovered for 3 h at 30°C in 1 mL liquid YPD. After recovery, cells were plated onto YPD plates with 100 μg/mL nourseothricin and incubated at 30°C for 2 days. Single colonies were picked and identified by PCR and sequencing with the primers listed in Table S1. The correct transformants containing I380L and Y682C mutations in *PDR1* were named BMU-*PDR1*^I380L^ and BMU-*PDR1*^Y682C^.

### Transcriptional profiling using RNA sequencing

*N. glabratus* cultures were inoculated into liquid YPD medium (initial OD_600_ of 0.1) and grown at 30°C, 200 rpm, to logarithmic phase (OD_600_ of 0.6-0.8). FLC was added to each culture at a concentration equivalent to the 0.5*MIC value of each strain (1 mg/L FLC for BMU-*PDR1*^WT^, 64 mg/L FLC for BMU-*PDR1*^I380L^ and BMU-*PDR1*^Y682C^) when the OD_600_ reached 0.3, then the cultures were allowed to grow until OD_600_ reached 0.6-0.8 (about 3 h). In parallel, control group without the exposure of FLC was performed. Total RNA was extracted using the Trizol reagent kit (Invitrogen, Carlsbad, USA) according to the manufacturer’s protocol. RNA-sequencing was performed using the Illumina Xten platform by Gene Denovo Biotechnology Co. (Guangzhou, China) as described previously [31]. Three biological replicates for each strain were sequenced. After filtering, *N. glabratus* ATCC2001 reference genome was mapped to the clean reads using HISAT2. Genes with FDR < 0.05 and fold change (FC) > 1.5 (log2-FC > 0.585) were considered as differentially expressed genes (DEGs). PCA was performed with R package gmodels (http://www.rproject.org/). Gene Ontology (GO) enrichment analysis was performed using Gene Ontology database (http://www.geneontology.org/).

## Results

### High rate of low-susceptibility to triazoles in clinical *N. glabratus* isolates

The 109 isolates of *N. glabratus* were collected from 20 cities in China during 2002–2021, predominantly isolated from blood (80, 73.4%) and ascitic fluid (14, 12.8%). Fifty three isolates (48.6%) were resistant/non-wild type (NWT) to triazoles, including 12, 4, 48 and 32 isolates resistant/NWT to fluconazole (FLC), itraconazole (ITC), voriconazole (VRC) and posaconazole (POS), respectively. Fifteen isolates were cross-resistant to VRC and POS, 8 to FLC, VRC and POS, and 4 to FLC, ITC, VRC and POS. The majority of isolates were susceptible to echinocandins except for 2 isolates with multidrug-resistance to echinocandins and POS, which harboured S663P substitution in *FKS2*, a mutation proven to confer echinocandin-resistance in *N. glabratus*, and 6 isolates were intermediate to caspofungin (CAS). All isolates were wild-type (WT) to amphotericin B (AMB) (Supplemental Dataset S1).

### Genome sequencing and gene annotation of *N. glabratus* isolates

The 109 *N. glabratus* genomes were sequenced with WGS, followed by de novo assembly and annotation (Supplemental Figure S1; Materials and Methods). The number of assembled contigs varied from 94 to 544, with a median N50 size of 402 Kb (Table 1; Supplemental Dataset S2). The total length of the assemblies varied from 12.21 to 13.72 Mb with a medium of 12.30 Mb, very close to that of the reference genome of *N. glabratus* strain CBS138 (12.34 Mb) (Supplemental Dataset S2). BUSCO analysis with the fungi-specific marker set demonstrated that the duplicated rate and the missing rate of the assemblies varied within 1.1% – 1.3% and 4.0% – 4.5%, respectively, suggesting the high completeness and low contamination of the assembled genomes (Supplemental Dataset S3). The contigs of each strain were also aligned to the reference genome of CBS138. The contigs could cover the 13 chromosomes and one mitochondrion genome evenly, with overall coverage varying from 99.38 to 99.78%, further confirming the high quality of the genome assemblies (Supplemental Figure S2; Supplemental Dataset S4). In total, 5087–5817 protein-encoding genes were predicted from the genomes with GeneMarker, among which 4797–5511 were functionally annotated or could be well mapped to known genes (Table 1; Supplemental Dataset S2). Generally, 98.3% (87.6%–98.9%) of the protein-encoding genes could be found with homologs from the CBS138 genome (Supplemental Dataset S5), while 96.4% (85.9%–97.1%) of the genes were mapped to the 5053 (5053/5258, 96.1%) eggNOG-annotated CBS138 genes (Supplemental Dataset S6). The results demonstrated that, the distribution of annotated and new genes in the 109 *N. glabratus* isolates was similar to that of the reference strain, and the species showed certain genome plasticity allowing for inclusion of new genes.

**Table 1.** Summary of the properties of Nakaseomyces glabratus genome assemblies.

Property	Median	Range
Number of genome assemblies	109	–
Genome size (megabases)	12.30	12.21–13.72
Contig N50 (kilobases)	402.30	42.80–709.69
Coverage of the reference genome (%)^1^	99.54	99.38–99.78
GC content	38.55	38.51–40.52
Number of predicted genes	5146	5087–5817
Genes with homology to known genes	4888	4797–5511
New genes	257	234–393

^1^ Reference strain and the genome accession: *Nakaseomyces glabratus* ASM254v2, NC_005967.2

**Figure 1.**
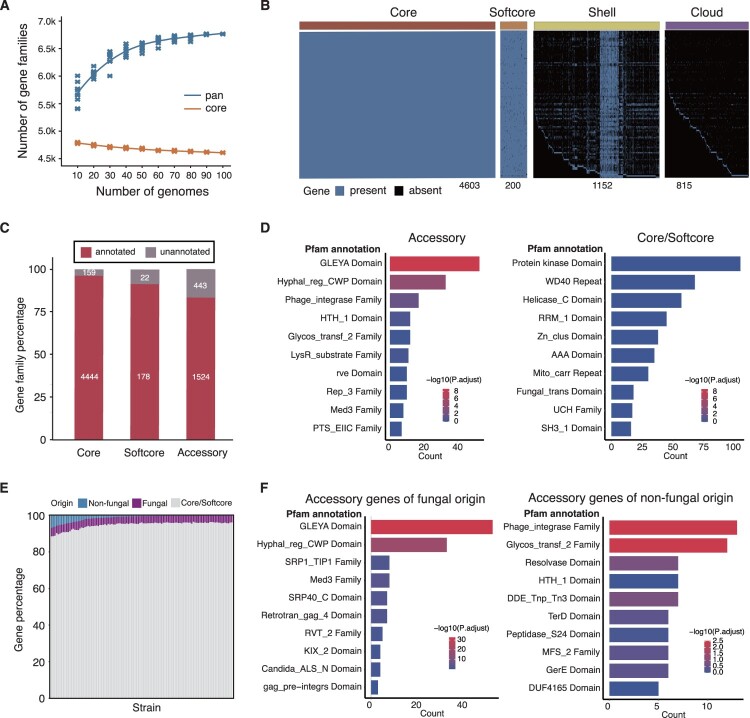
The pan-genome of the *Nakaseomyces glabratus* isolates. (A) The relationship between the number of genomes and that of core- and pan-genome families. With the increasing number of *N. glabratus* genomes included, the number of pan-genome families were increased while the number of core-genome families were decreased. (B) The matrix of pan-genome families identified in *N. glabratus* genomes. The pan-genome was subdivided into core (present in all isolates), softcore (present in >95% of the isolates), shell (present in 5–95% of the isolates) and cloud (present in <5% of the isolates) genomes. (C) The number and percentage of core, softcore and accessory genes. Compared with core or softcore genome, the accessory genome contained a lower proportion of genes functionally annotated. (D) Pfam enrichment analysis of accessory genome and core/softcore genome. The accessory genome predominantly enriched in genes encoding hyphally regulated cell wall proteins, GLEYA-containing proteins. The core/softcore genome predominantly enriched in genes encoding proteins with kinase domain, WD40 repeat domain, helicase conserved C-terminal domain. (E) Percentage of fungal and non-fungal original genes in accessory genome. In each individual of the 109 isolates, the number of fungal original genes was similar while that of non-fungal original accessory genes was various. (F-G) Pfam enrichment analysis of fungal and non-fungal original genes in accessory genome. Fungal original genes were enriched in those encoding GLEYA-containing proteins and hyphally regulated cell wall proteins (F), while non-fungal original genes were enriched in phage_integrase family and Glycosyltransferase family 2 (G).

Totally, 6770 pan-genome families (distributed in at least two isolates) were identified from the 109 genomes, and the number of families turned to saturation when 100 and more isolates were included (Figure 1(A); Supplemental Dataset S7). The pan-genome was composed of 4603 core (68.0% of the pan-genome), 200 softcore, 1152 shell and 815 cloud gene families (Figure 1(B)), where the core, softcore, shell and cloud gene families define those distributed in all, 95%−100%, 5%−95% and < 5% of the isolates respectively. The large accessory genome (1967 shell and cloud gene families, 29%) and also the large percentage of cloud gene families (815/6770, 12%) further confirmed the high plasticity of *N. glabratus* genomes (Figure 1(B); Supplemental Figure S3). Compared with core genome, the accessory genome contained a lower proportion of genes functionally annotated (Figure 1(C)), which predominantly enriched in GLEYA-containing proteins, hyphally regulated cell wall proteins and phage integrase proteins (Figure 1(D)).

### Highly plastic *N. glabratus* genome driven by both gene amplification and horizontal transfer

Around 83–106 pan-genome families in each *N. glabratus* isolate have experienced gene amplification, among which 9–19 pan-genome families have experienced multiple rounds of amplification (Supplemental Dataset S8). Consistent with observations from the accessory genome, both the amplified and multi-round-amplified gene families were enriched with GLEYA-containing proteins and hyphally regulated cell wall proteins (Supplemental Figure S4). The results were also consistent with one previous study, which reported extensive copy number variation of genes, especially cell wall-associated genes, in *N. glabratus* [32]. We also found a tandem repeat of ∼ 60 Kb chromosome A segment containing 16 genes in one isolate (Supplemental Dataset S9), which was validated experimentally (data not shown).

Besides gene duplication, the individual *N. glabratus* isolates also showed amplification of genes with lower homology to the background gene families. These gene families were within the accessory genome, which could have been acquired by horizontal gene transfers (HGT) from fungal or even non-fungal origins. The non-fungus-originated genes did not appear to be caused by contamination, since all the analysed gene families were detected at least in two different *N. glabratus* isolates and more than 64.1% of the families were distributed in three or more isolates (Supplemental Dataset S10). Many non-fungus-originated genes were also located in the contigs with long continuity (Supplemental Dataset S11). In each isolate, a relatively stable proportion of the accessory genes were obtained from other fungi (Figure 1(E)). Consistent with the observation for all gene families from the accessory genome, the fungus-originated accessory genes were enriched in those encoding GLEYA-containing proteins and hyphally regulated cell wall proteins (Figure 1(F)). In contrast to the fungus-originated genes, the isolates did not contain an even number of non-fungus-originated accessory genes (Figure 1(E)). Except for some isolates, most others contained only a few accessory genes of non-fungal origin (Figure 1(E)). The non-fungus-originated genes also showed functional enrichment for phage integrase and glycosyltransferase, but the significance was much smaller than those functions enriched in fungus-originated genes (Figure 1(F)).

Taken together, *N. glabratus* shows a high genome plasticity with apparent gene variation. The genome is characterized by extensive gene duplications and the large accessory gene families that could potentially be acquired by HGT events. Genes that vary most typically encode GLEYA domains and cell wall associated proteins, which are frequently duplicated or acquired from other fungi.

### Genomic signatures linked to drug resistance

**Figure 2.**
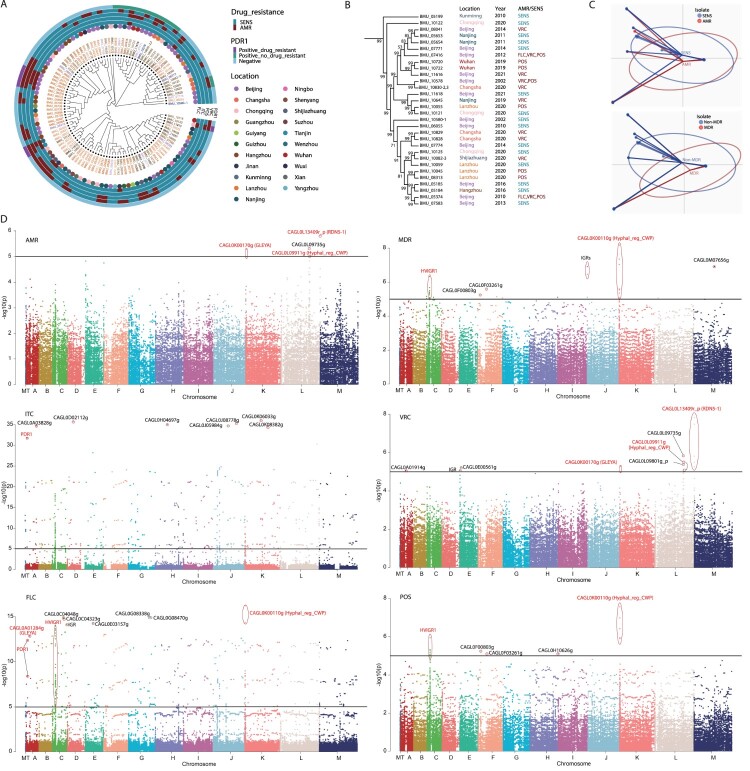
Associations between drug resistance and genomic variations in *Nakaseomyces glabratus*. (A) Phylogenic tree based on core-genome of *N. glabratus* isolates. The triazole-resistant isolates and their geographical locations exhibited a scattered distribution on the phylogenic tree. The metadata rings outside of the tree indicated the triazole-resistant phenotype. Mutations in the *PDR1* gene were indicated on the outside of the tree. The names of isolates for the same year were highlighted in the same colour. SENS, sensitive; AMR, anti-microbial resistant; VRC, voriconazole; POS, posaconazole; ITC, itraconazole; FLC, fluconazole. (B) A robust cluster from the phylogenetic tree. The triazole-resistant isolates and their geographical locations and isolation years exhibited a scattered distribution on the subtree. The percentages of bootstrapping tests were shown for each node. (C) Primary component analysis (PCA) of amino acid composition in polymorphic sites in core genes. The isolates were classified into AMR and SENS (up), or multi-drug resistant (MDR) and non-MDR groups (down). The non-overlapping of the PCA scatterplot indicated atypical genomic difference between AMR and SEN isolates (up), and between MDR and non-MDR isolates (down). (D) The associations between drug resistance and genome-wide SNPs showed on Manhattan diagrams. The significantly resistance-related SNPs located in protein-coding genes or intergenic regions were highlighted. The significance cutoff was preset as *P*-value < 1e-05.

In order to find out genomic signatures linked to drug-resistance, we performed a series of genome association analyses. Firstly, phylogenetic analysis of *N. glabratus* isolates based on core-genome SNPs showed that drug resistance had no typical associations with the evolutionary lineages (Figure 2(A)). Consistently, a representative robust subtree with 30 strains showed that the drug resistance was unrelated with the phylogeny, isolation places and time (Figure 2(B)). Despite local clustering for a few isolates according to the isolation place or time, there is a lack of overall clustering for the drug resistance phenotype of the isolates associated with isolation place or time (Figure 2(A)). For example, two strains isolated from Changsha in 2020, BMU10828 and BMU10829, were closely clustered, but they were much closer to the strain BMU07774 isolated from Beijing in 2014 than another strain BMU10830-2-3 isolated from Changsha in 2020 (Figure 2(B)). The four strains, BMU11618, BMU10645, BMU10055 and BMU10121, were clustered robustly, but they were isolated from four different regions of China in three different years, with different antifungal resistance profiles (Figure 2(B)). A GLM was fit for the resistance phenotype to each antifungal, with phylogenetic branch, isolation place and isolation year as covariates, and none of the covariates was found to significantly correlate with the resistance phenotype (FDR > 0.2 for all the covariates), further demonstrating that phylogenetics, isolation place and isolation time had little association with the antifungal resistance. However, Principal Component Analysis (PCA) with core-genome SNPs revealed that the anti-microbial resistant (AMR) and sensitive (SEN) isolates showed atypical difference, which was more significant between multi-drug resistant (MDR) and non-MDR isolates (Figure 2(C)), suggesting the existence of possible association between resistant phenotype and genetic variations.

Further, we performed GWAS to detect possible genomic signatures associated with drug-resistance. Resistance-related signatures were located in protein-coding genes or intergenic regions, including *PDR1* associated with FLC- and ITC-resistance, a highly variable intergenic region (HVIGR1) in chromosome C associated with FLC- and POS-resistance, cell wall protein genes such as *AWP2* (CAGL0K00110 g) associated with FLC- and POS-resistance, and notably numerous genes encoding proteins with GLEYA domains such as *EPA10* (CAGL0A01284 g) associated with FLC-resistance and *EPA22* (CAGL0K00170 g) with POS-resistance (Supplemental Dataset S12, Figure 2(D)).

### Signature-based machine-learning models predict drug-resistance and identify potential resistance determinants

**Figure 3.**
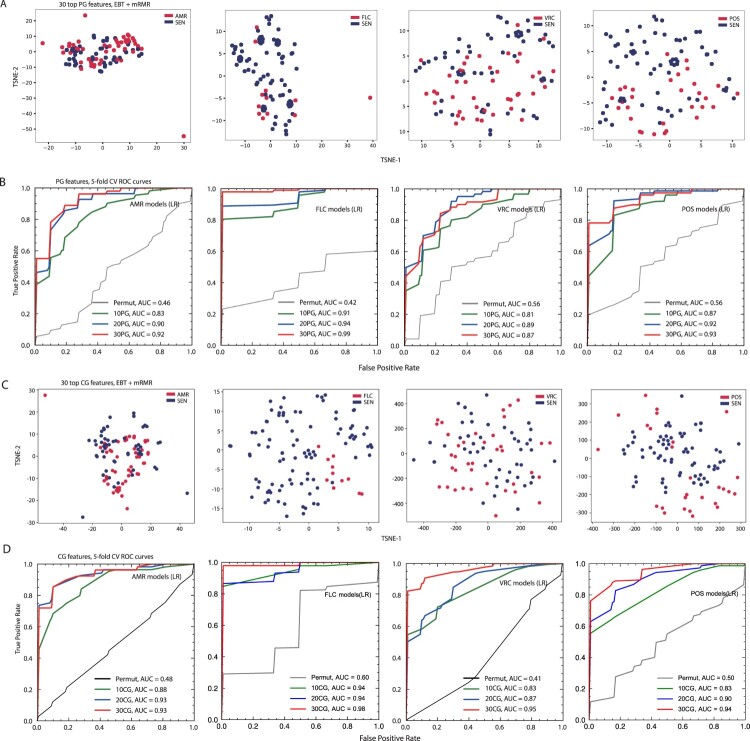
Predicting drug-resistance by machine-learning models based on pan- and core-genome signatures. (A) T-SNE clustering of *N. glabratus* isolates with various antifungal-susceptibility profiles using pan-genome (PG) signatures. The majority of the resistant strains can be distinguished from the SEN ones by the PG signatures. AMR, anti-microbial resistant; FLC, fluconazole; VRC, voriconazole; POS, posaconazole. (B) Receiver operating characteristic (ROC) curves of Logistic Regression (LR) models based on PG signatures. The LR models based on 30 PG signatures exhibited excellent capability to identify resistant isolates. Permut, permutation model based on 30 PG signatures. (C) T-SNE clustering of *N. glabratus* isolates with various antifungal-susceptibility profiles using core-genome (CG) signatures. The majority of the resistant strains could be distinguished from the SEN ones by the CG signatures. (D) ROC curves of LR models based on CG signatures. The LR models based on 30 CG signatures exhibited excellent capability to identify resistant isolates. Permut, permutation model based on 30 CG signatures.

Since abundant genomic signatures associated with azole resistance were detected by GWAS as above mentioned, we tried to propose a new feature-selection strategy combining pan-genome-based, per-site, supervised rate comparison, and mRMR-based redundancy filtering to narrow down the features and facilitate the detection of more relevant signatures. Subsequently, these signatures were applied to train multiple machine-learning (ML) models to distinguish AMR strains from the SEN ones (Methods; Supplemental Dataset S13). The majority of the AMR strains could be distinguished from the SEN ones by the signatures as demonstrated by T-SNE analysis (Figure 3(A)). Among various ML models, the top 30 signature-trained LR models exhibited the best capability of resistance prediction with the average rocAUC reaching 0.92, 0.99, 0.87 and 0.93 in prediction of resistance to any drug, FLC, VRC and POS, respectively (Figure 3(B); Supplemental Dataset S14). We named the above strategy ML-based feature selection (MLFS) strategy (Supplemental Figure S5). By applying this MLFS strategy, pan-genome signatures that correlate significantly to resistance were identified. Among these pan-genome signatures, several that located in genes encoding hyphally regulated cell wall proteins or proteins containing GLEYA domains were also detected by GWAS. Notably, *RTT109*, the homolog of which encodes histone acetyltransferase and regulates *ERG11* expression in *C. albicans* [33], was identified as the FLC-resistant signature.

Although those pan-genome-based models displayed excellent capability in predicting drug resistance, pan-genome signatures may not exist in every individual of the corresponding organism. Therefore, we applied a similar MLFS strategy to select core-genome signatures. Consequently, the most relevant core-genome features that distinguish AMR strains from the SEN ones were identified (Supplemental Dataset S15; Figure 3(C)). Similarly, the top 30 signature-trained LR models exhibited the best capability of resistance prediction with rocAUC of 0.93, 0.98, 0.95 and 0.94 in the prediction of resistance to any drug, FLC, VRC and POS, respectively (Supplemental Dataset S16; Figure 3(D)). Notably, this core-genome MLFS strategy identified several signatures that located in resistance-mediating genes, including *PDR1*, *FPS1* encoding glycerol transporter which involves flucytosine- and echinocandin-susceptibility [34], and *AHC1* encoding histone acetyltransferase which influences triazole-susceptibility [35]. These results suggested that MLFS strategy could not only accurately predict resistance but also effectively identify underlying resistance determinants. We also annotated the SNPs of genes that were reported to be associated with azole resistance, including *ERG11*, *SNQ2*, *CDR1* and *CDR2* (Supplemental Dataset S17). Models based on these gene polymorphisms as features could predict the AMR, MDR, FLC, POS or other resistance well, with the performance (rocAUC: 0.54∼0.66) much poorer than those of core-genome models (Supplemental Figure S6).

### The mutations I380L and Y682C in PDR1 confer triazole-resistance in *N. glabratus*

**Figure 4.**
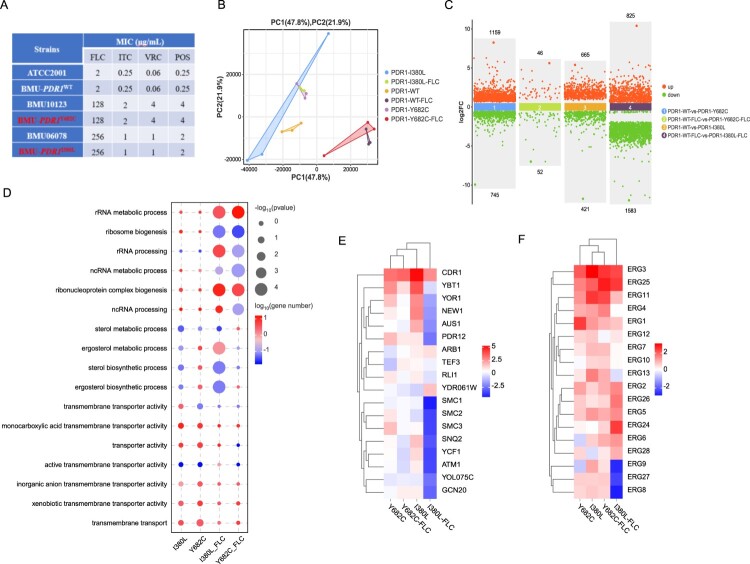
Transcriptome analysis of *Nakaseomyces glabratus PDR1* mutant. (A) Antifungal susceptibilities of *N. glabratus* isolates to fluconazole (FLC), itraconazole (ITC), voriconazole (VRC), and posaconazole (POS). MIC, minimum inhibitory concentration. BMU-*PDR1*^I380L^ and BMU-*PDR1*^Y682C^ were resistant to triazoles with MICs identical to clinical isolates BMU06078 and BMU10123, which habor I380L and Y682C mutations in *PDR1*, respectively. (B) Principal component analysis (PCA) based on normalized RNA-sequencing read counts. The three biological replicates per strain displayed good correlation and reproducibility. PDR1-I380L and PDR1-Y682C represented *PDR1* mutant strains BMU-*PDR1*^I380L^ and BMU-*PDR1*^Y682C^, and PDR1-WT represented *PDR1* wild-type (WT) control strain BMU-*PDR1*^WT^; PDR1-I380L-FLC, PDR1-Y682C-FLC, and PDR1-WT-FLC represented the corresponding strain with exposure to fluconazole (FLC). (C) Number of differentially expressed genes (DEGs) of *PDR1* mutant. Compared with WT control, *PDR1* mutants exhibited hundreds to thousands DEGs with or without exposure to FLC. DEGs were defined as genes with a false discovery rate (FDR) < 0.05 and fold change (FC) > 1.5 (log2-FC > 0.585). (D) The Gene ontology (GO) enrichment analysis of DEGs. Significant enrichment of DEGs involved in RNA processes, ergosterol processes, and transporter activity in BMU-*PDR1*^I380L^ and BMU-*PDR1*^Y682C^. -Log10-*P* values were size-coded, and Log10-gene numbers were colour-coded. (E-F) Heat map of DEGs encoding drug efflux pumps and ergosterol biosynthesis processes enzymes in *PDR1* mutant. Compared to BMU-*PDR1*^WT^, genes encoding drug efflux pumps involved in triazole-resistance such as *CDR1* (E), and the genes encoding ergosterol biosynthesis processes enzymes including *ERG1*, *ERG3*, and *ERG11* (F), were upregulated in BMU-*PDR1*^I380L^ and BMU-*PDR1*^Y682C^. Log2-fold change (FC) expression values were colour-coded.

Several mutations in *PDR1*, including G346D and G1099D, have been confirmed to confer triazole-resistance [36]. These mutations, together with other SNPs, were identified by both GWAS and MLFS strategies in our study (Figure 2D; Supplemental Dataset S5,8). In order to investigate whether the mutations I380L and Y682C confer triazole-resistance, we constructed the mutant strains BMU-*PDR1*^I380L^ and BMU-*PDR1*^Y682C^ via CRISPR-Cas9 system. Antifungal susceptibility tests showed that BMU-*PDR1*^I380L^ and BMU-*PDR1*^Y682C^ were resistant to triazoles with MICs identical to those against clinical isolates with these mutations (Figure 4(A); Supplemental Dataset S1).

Subsequently, the global transcriptional profiles were assessed in BMU-*PDR1*^I380L^ and BMU-*PDR1*^Y682C^ with or without exposure to FLC (Figure 4(B,C)). The Gene Ontology (GO) enrichment analysis of differently expressed genes (DEGs) revealed significant enrichment of genes involved in RNA processes, ergosterol processes, and transporter activity in BMU-*PDR1*^I380L^ and BMU-*PDR1*^Y682C^ (Figure 4(D)). Compared to BMU-*PDR1*^WT^, several genes encoding drug efflux pumps involved in triazole-resistance, such as *CDR1*, *YBT1*, and *YOR1* (Figure 4(E)), and the genes encoding ergosterol biosynthesis processes enzymes including *ERG1*, *ERG3*, and *ERG11* (Figure 4(F)) were upregulated in BMU-*PDR1*^I380L^ and BMU-*PDR1*^Y682C^. These indicated that the I380L and Y682C mutations in *PDR1* resulted in triazole-resistance via upregulating the downstream genes encoding drug efflux pumps and ergosterol biosynthetic process enzymes.

## Discussion and conclusion

The WGS on 109 clinical isolates of *N. glabratus* revealed that the average size of the genome assembly was 12.3 Mb, consistent with that of the reference strain (12.3 Mb, ASM254v2). A total of 6770 pan-genome families, comprising 4803 core – and softcore – genome families (70.9%) and 1967 accessory-genome families (29.1%), were identified. The genome assemblies exhibit a high coverage of the reference *N. glabratus* genome and reach the chromosome level with an average coverage of 99.54% across the 13 chromosomes and the mitochondrion chromosome, comparable to those described by Marcet-Houben et al. [8]. However, a higher proportion of core-genome (94%) and a lower proportion of accessory-genome (6%) in *N. glabratus* were described in their study [8]. We speculate that this discrepancy is probably due to the small sample size of only 21 isolates, which insufficiently representing species diversity. Using the same method as described in our study here, we further performed pan-genome analysis on an additional dataset comprising 91 genomes from the NCBI SRA database. Consequently, we observed a comparably high proportion of accessory-genome similar to those derived from 109 isolates analysed here (data not shown). Peter et al. described that *S. cerevisiae*, which is closely related to *N. glabratus*, exhibited a similar proportion of accessory-genome (15%−36%) [37,38]. In other human pathogenic fungi such as *C. albicans*, *Cryptococcus neoformans* and *Aspergillus fumigatus*, similarly, the accessory-genome comprises around 10%−30% of the pan-genome [38,39]. Generally, *N. glabratus* exhibits a large percentage of accessory-genome which is considered to be more unstable under selective pressure, indicating the active evolutionary dynamics in genome [37]. The characteristic of extended accessory-genome in *N. glabratus* may provide the genomic basis for the large phenotypic diversity of the organism.

Acquisition of the accessory-genome can occur through duplication or HGT, the latter of which is recognized as a major driver of genome evolution in bacteria and plays a crucial role in the widespread dissemination of AMR. In fungi, intrakingdom HGT events account for up to 8% of accessory-genome [38], yet have rarely been reported in *N. glabratus*. However, an expanded accessory-genome in *N. glabratus*, which was acquired by HGT from fungal and non-fungal origins, was identified in our study. Genes originating from fungi were enriched in genes encoding GLEYA-containing proteins and hyphally regulated cell wall proteins, predominantly belonging to the adhesin family. Nearly two-thirds of adhesin genes in *N. glabratus* are located within subtelomeric regions which are thought to be dynamic parts of the genome, exhibiting substantial divergence [40]. This genetic characteristic makes the cell wall of *N. glabratus* vary in adhesin content depending on the environment [8]. Therefore, the significant content of adhesin or adhesin-like genes in the accessory genome of *N. glabratus* isolates probably governs the organism’s adaptation and adhesion to host surfaces, the first step of pathogenicity in *N. glabratus*. The current study disclosed the large amplification and variation of adhesin genes in *N. glabratus*, but more extensive experiments are desired to explore the exact biological roles of these genes and their variation.

Besides the genes of fungal origins, in total, we captured 6959 non-fungus originated genes from the 109 *N. glabratus* isolates, spanning 1117 accessory pan-genome families (Supplemental Dataset S10). Except for a few isolates with a larger percentage, the non-fungal genes in most of the *N. glabratus* strains cover around 1% of the whole genome-encoding genes (Figure 1(E)). The non-fungal accessory families were repeatedly identified from multiple isolates (all from > =  2 isolates, 64.1% from > =  3 isolates and 36.5% from > =  5 isolates; Supplemental Dataset S10), while the genes were often located in contigs with a long continuity (Supplemental Dataset S11), largely excluding the influence caused by possible contamination. Although most of the genes could be found with homologs in the CBS138 genome, the homology was commonly low, but non-fungal gene hits were with large homology (Supplemental Dataset S10), excluding possible mis-annotations. The genes of non-fungal origins, mainly from bacteria, encode proteins with diverse functions. Notably, enrichment of phage integrases and glycosyltransferases was observed. The most fundamental biochemical function of phage integrase genes in bacteria is catalyzing site-specific recombination and is the key vector for HGT, acting as a “genetic engineer” driving bacterial evolution and spreading virulence and resistance genes [41,42]. The core function of glycosyltransferase genes is to encode enzymes that catalyze glycosylation reactions, involving cell wall biosynthesis, capsular polysaccharide and exopolysaccharide synthesis, lipopolysaccharide synthesis, protein glycosylation, and antibiotic modification and resistance. The biological significance in microorganisms include virulence and pathogenesis, stress adaptation, and drug resistance [43]. Therefore, we assumed that phage integrases and glycosyltransferases genes observed in *N. glabratus* might play a key role in HGT and antifungal resistance, whereas the exact role needs to be further explored. Some bacterial transporter genes, including garP and mdtG, which contribute to bacterial AMR [44,45], were detected in several triazole-resistant isolates of *N. glabratus*. GarP and mdtG encode carbohydrate transporter and major facilitator superfamily transporter in *E.coli*, which are involved in host adaption and multidrug resistance. These bacterial-origin genes may transfer to *N. glabratus* and express drug efflux pump proteins, resulting in triazole-resistance of *N. glabratus*. The way of the acquisition of these genetic materials in *N. glabratus* remains unclear. Previous studies have revealed that the transmission of genetic materials, including HGT occur between fungi and other microorganisms when contact with each other, such as *Agrobacterium tumefaciens* [46] and *A. fumigatus* [47]. In addition, intracellular organisms, such as mycoviruses in plant pathogenic fungi and endosymbiotic bacteria in *Rhizopus* spp. [48], could influence the virulence or other phenotypes of the host fungi, regardless of these exogenous genetic materials integrate in the chromosome or not. In *N. glabratus*, some of these exogenous genetic materials from fungal or non-fungal origins could possibly be selected out to generate genetic diversity under specific stress for stress adaptation and shaping genome plasticity in *N. glabratus*. However, the contribution of these exogenous genes to phenotypes including drug-resistance need to be further elucidated.

Aneuploidy and gene copy number variation (CNVs) are major mechanisms for adaptation in asexual *Candida* species. In *C. albicans*, nearly 50% of FLC-resistant isolates carry aneuploidy, particularly in chromosome 5 where *ERG11* and a transcription factor *TAC1* activating *CDR1* and *CDR2* expression are located [49]. In *C. tropicalis* [50] and *C. parapsilosis* [51], extra copies of *ERG11* due to the duplication of the gene-coding region rather than aneuploidy are commonly observed in triazole-resistant clinical isolates. In *C. auris*, an additional copy of chromosome 5 containing *ERG11* and *TAC1* has been observed in experimentally-induced triazole-resistant isolates [52]. It was reported that about 3% clinical isolates of *N. glabratus* diploid or hyperdiploid [53], however, the contribution of which to drug-resistance remains ambiguous. Aneuploidy of chromosome E encompassing *ERG11* was commonly detected in experimentally-induced triazole-resistant *N. glabratus* isolates [54]. In this study, no diploid or hyperploid was observed in 109 clinical isolates, different from other *Candida* pathogens as well as previously described *N. glabratus*. Additionally, large genomic duplications were not identified except for a tandem repeat of ∼ 60 Kb chromosome A segment being observed in only one triazole-resistant isolate. This segment harbours 16 genes rather than *ERG11* or the transcription factor *PDR1*, two prominent factors driving triazole-resistance in *N. glabratus*. The role of these duplicated genes needs to be further investigated. We hence speculate that genome plasticity of *N. glabratus* is constructed by expanded accessory-genome, driven from HGT, rather than by aneuploidy or CNVs.

However, the precise genomic structural characteristics, have failed to be comprehensively elucidated to be linked to phenotypes, especially antifungal resistance, by the global pan-genome analysis. Therefore, we conducted genome-wide association analyses to identify genomic clues that significantly correlate with drug-resistance. First, phylogenetic analysis based on core-genome SNP revealed no evolutionary lineages related to drug resistance. However, PCA revealed atypical genetic differences between sensitive and resistant isolates, particularly MDR isolates, indicating a general association between resistant phenotype and genomic diversity. Hence, we performed GWAS and identified numerous nucleotide polymorphisms significantly associated with drug resistance. Notably, several mutations in *PDR1*, a crucial transcription factor regulating the expression of genes involved in triazole-resistance, were identified to be linked to ITC- and FLC-resistance, including the mutations Y682C and I380L which were further confirmed to confer triazole-resistance in this study. Arastehfar et al. reported one susceptible isolate harboured S76P, V91I, L98S, T143P, I380L, K704N mutations simultaneously in *PDR1* [55]. However, in our study, the azole-resistant isolate harboured a single mutation I380L in *PDR1*, and further validated to confer resistance through gene-editing. We speculate the deviation may be due to the impact of other mutations combined with the I380L mutation, while it is known that multiple mutations in a protein can give rise to complex conformational changes that impact its functionality. Nevertheless, the precise effects of the I380L mutation and other concomitant mutations on the structure and function of *PDR1* require further experimentation, including genetic manipulation, structural analysis, and functional assays, to be fully elucidated. Additionally, lots of genes encoding GLEYA-containing proteins were identified to be linked to drug-resistance. These genes play a significant role in virulence and protecting fungal cells from harmful stress [56], indicating potential avenues for pathogenicity and adaptation to stresses in *N. glabratus*. By using the above-described strategy of genetic and drug-resistant association analysis, numerous candidate genes potentially conferring drug resistance have been identified in this study. However, the contribution of these signatures in candidate genes to drug-resistance needs to be further confirmed by labour-intensive and time-consuming studies.

Therefore, we further developed a novel MLFS strategy to narrow down the features so as to identify more relevant signatures for directing subsequent experiments, by which the resistance determinants would be identified. ML approaches have rarely been applied directly in identification of fungal resistance mechanisms till date. Most other similar studies are based on comparison of the genomes or transcriptomes to identify the resistance-associated markers, followed by experimental validations. However, there could be too many signature candidates, most of which could be filtered by MLFS due to the low relevance or large redundancy. Besides typical features, MLFS could also identify relevant but atypical resistance-associated marker genes that could not be detected by those traditional comparative-omics based strategies. Moreover, MLFS has a potential-specific advantage that it can capture the linear or nonlinear relationship among multiple marker genes that may interact with each other, contributing to resistance synergistically. The strategy showcased in their study can be used to narrow down the possible genetic variants leading to a resistant phenotype. Using this MLFS strategy, we have identified several pan-genomic signatures in genes including *RTT109* and core-genome signatures in genes including *PDR1*, *FPS1* and *AHC1*, which were proven to confer drug-resistance in previous studies. By using CRISPR-based gene editing technology and RNA-sequencing, we also demonstrated that the novel mutations identified by the MLFS approach, I380L and Y682C in *PDR1*, confer triazole-resistance by upregulating the genes encoding drug efflux pumps and ergosterol biosynthetic processes enzymes (Figure 4). These data confirmed that the MLFS strategy could effectively assist in identifying the potential resistance determinants.

Besides identifying resistance mechanisms, the ML models trained in the MLFS approach could also accurately predict new antifungal-resistant isolates. Based on the selected genomic signatures, multiple ML models were trained to distinguish drug-resistant strains from the – susceptible ones. The top 30 signature-trained LR models, regardless of core- or pan-genome signatures, exhibited excellent capability for predicting antifungal drug resistance with rocAUC exceeding 0.9 in any drug, FLC, POS, and VRC. Due to the rare number of ITC-resistant isolates, a 5-fold cross-validation assessment could not be performed, so that the models predicting ITC-resistance have not been established. It should be noted that, besides pan-genome and core-genome signatures, we also tested other features, such as gene copy number. Models based on the combinatory pan-genome and copy number features, i.e. ePG models, were trained and evaluated for the performance. However, the performance was not better than the pan-genome models according to the 5-fold cross-validation assessment (Supplemental Figure S7), so we did not include the ePG models in the main results. However, inclusion of these and more other features in the models could possibly improve the prediction performance and therefore deserves to be further explored in future studies.

Although the ML models established in this study based on the pan-genome and core-genome signatures demonstrated superior performance in resistance prediction of *N. glabratus* isolates, the models need to be validated with more external datasets before practical application. There are very few *N. glabratus* isolates with both available genome sequences and comprehensive documentation about their antifungal resistance; therefore, we could not annotate an independent dataset to validate the model performance at present. However, the validation work is ongoing. In a preliminary analysis on 5 FLC, 4 VRC and 1 POS-resistant *N. glabratus* isolates, the ML models could correctly recall nearly all of them. However, more resistant and susceptible isolates are required to make more objective evaluation of the model performance. It should be pointed out that all the 4 isolates resistant to ITC in this study, are also resistant to other azoles, and therefore, the possible biases could not be excluded.

The models developed in the current study were only applicable to resistance prediction of azoles rather than echinocandins. In future, more clinical isolates with echinocandin resistance should be collected to train ML models predicting the isolates resistant to echinocandins. In fact, we also made comparative genomic analysis between the very limited number of, phylogenetically close, *N. glabratus* isolates resistant and susceptible to echinocandins. There are three isolates, BMU10720, BMU10722 and BMU11616, closely clustered in the phylogenetic tree (Figure 2(B)), but with the former two resistant and the latter one susceptible to echinocandins (Supplemental Dataset S1). Comparative analysis disclosed 61 and 60 pan-genome families specific to echinocandin-resistant and echinocandin-susceptible isolates, respectively (Supplemental Dataset S19). The families specific to echinocandin-resistant and echinocandin-susceptible isolates are both enriched in GLEYA domain containing proteins (Supplemental Figure S8). Two and seven pan-genome families are specifically amplified in echinocandin-resistant and echinocandin-susceptible isolates, respectively, many of which encode GLEYA domain-containing proteins or cell wall proteins (Supplemental Dataset S19; Supplemental Figure S8). In total, 208 consensus SNPs and INDELs that could alter the amino acids were disclosed between echinocandin-resistant and echinocandin-susceptible isolates, but no function was significantly enriched for these genes (Supplemental Dataset S19). The results could give some clues about the genetic differences responsible for echinocandin resistance. However, besides echinocandin, these three isolates also show different resistance to other antifungals, such as POS and VRC (Figure 2(B)). Therefore, the genetic variations identified by comparative genomic analysis could be biased to explain the echinocandin resistance. More isolates with strict stratification and corrections for resistance to other drugs are required to make more reliable observations for the mechanisms of echinocandin resistance.

In conclusion, this study provides a comprehensive view of the plastic genome of *N. glabratus* comprising an expanded accessory-genome including non-fungal originated genes. Genomic signatures associated with drug-resistance were identified, which were further applied to develop the MLFS strategy to predict antifungal-resistance and identify potential resistance determinants. These will provide direction for experimental research to shed light on novel antifungal-resistant mechanisms in *N. glabratus*. In addition, the MLFS strategy is anticipated to identify the genomic clues linked to phenotypic characteristics including antifungal-resistance in pathogenic fungi.

## Author contributions

W. L., Q.W. and Y.W. conceptualized and designed the study. Q.W. and Z.W. carried out the experimental work. X.T. and Y.L. performed sequencing. Y.W., R.C., X.C., H.Z., J.Y. and X.W. carried out the extensive bioinformatics and analysed the data and interpreted the results. Q.W. and Y.W. wrote the primary manuscript and all authors were involved in the editing and reviewing the manuscript. W.L. acquired the primary funding for this work.

## Supplementary Material

Dataset_S17_Models_with_azole_resistance.xlsx

Supplemental Dataset S19.xlsx

Dataset_S12_GWAS.xlsx

Dataset_S8_Copy_number_varied_pan_genome_families.xlsx

Supplemental Dataset S18.xlsx

Supplemental Table S1_for reviewers only.xlsx

Dataset_S13_Pan_genome_AMR_features.xlsx

Fig_S5.pdf

Supplemental_Materials-clean.docx

Dataset_S16_CG_models_performance.xlsx

Dataset_S11_Non_fungal_accessory_genes_genome_continuity.xlsx

Dataset_S15_Core_genome_AMR_features.xlsx

Dataset_S10_Non_fungal_accessory_genes.xlsx

Dataset_S9_Large_duplication_in_BMU05374.xlsx

Fig_S7.pdf

Fig_S1_Pipeline.pdf

Fig_S4.pdf

Fig_S8.pdf

Dataset_S4_Contigs_mapped_to_CBS138_genome.xlsx

Fig_S3_revised.pdf

Dataset_S3_BUSCO_analysis.xlsx

Fig_S2.pdf

Dataset_S14_PG_models_performance.xlsx

Dataset_S7_Pan_genome.xlsx

Dataset_S1_Isolate_drug_susceptibility.xlsx

Dataset_S5_Proteins_mapped_to_CBS138_proteins.xlsx

Fig_S6.pdf

Dataset_S2_Assembly_statistics.xlsx

Description_on_supplemental_materials.docx

Dataset_S6_Proteins_mapped_to_CBS138_annotated_proteins.xlsx
