# Supplementary material for: Genome analysis and machine learning-based feature selection strategy reveal potential drug-resistance determinants in Nakaseomyces glabratus
Source: Emerg Microbes Infect. 2025 Dec 13;14(1):2595789. doi: 10.1080/22221751.2025.2595789 (PMC12704144; doi:10.1080/22221751.2025.2595789)
Supplement: Fig_S5.pdf [file TEMI_A_2595789_SM5795.pdf]

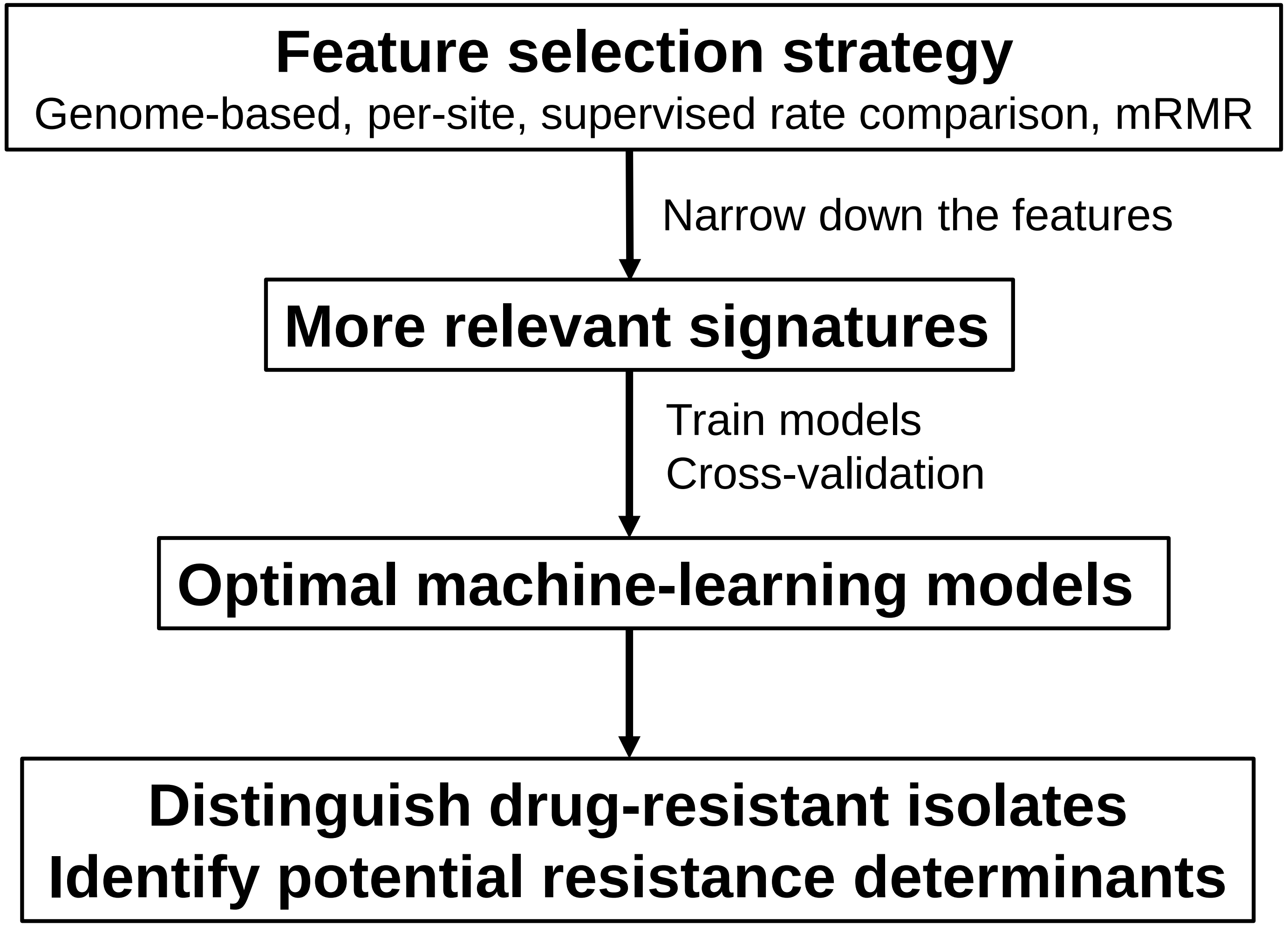
Feature selection strategy
Genome-based, per-site, supervised rate comparison, mRMR
Narrow down the features
More relevant signatures
Train models
Cross-validation
Optimal machine-learning models
Distinguish drug-resistant isolates
Identify potential resistance determinants
