# Supplementary material for: Genome analysis and machine learning-based feature selection strategy reveal potential drug-resistance determinants in Nakaseomyces glabratus
Source: Emerg Microbes Infect. 2025 Dec 13;14(1):2595789. doi: 10.1080/22221751.2025.2595789 (PMC12704144; doi:10.1080/22221751.2025.2595789)
Supplement: Supplemental_Materials-clean.docx [file TEMI_A_2595789_SM5794.docx]

Genome analysis and machine learning-based feature selection strategy reveal potential drug-resistance determinants in *Nakaseomyces glabratus*

**Supplemental Materials**

**Supplemental Dataset S1. The isolation and antifungal susceptibility information of *N. glabratus* isolates analyzed in this study.**

In total, 109 *N. glabratus* strains were isolated from more than 20 cities between 2002 and 2020. The antifungal susceptibility testing was performed on these isolates and documented in Dataset S1. Before genome analysis, the species was confirmed by PCR amplification and sequencing for the species-specific ITS region and 28S ribosomal subunit (D1/D2). The primers used for species identification and specific mutation detection (for later analysis) were also included in Dataset S1.

**Supplemental Figure S1. Research design.**

The 109 *N. glabratus* isolates were inoculated, cultured, followed by extraction of the genomic DNA and sequencing. After strict quality control for the reads, the genomes were assembled, assessed and annotated. Comparative genomic analysis was performed to observe the evolutionary properties of *N. glabratus* genomes, before identification of the genomic signatures associated with antifungal resistance of the isolates. Both traditional comparative genomic analysis and machine learning-based strategies were adopted to identify the genomic features most relevant to the antifungal (especially azole) resistance of *N. glabratus*. Experiments were also performed to validate the resistance contribution of newly identified antifungal-associated signatures.


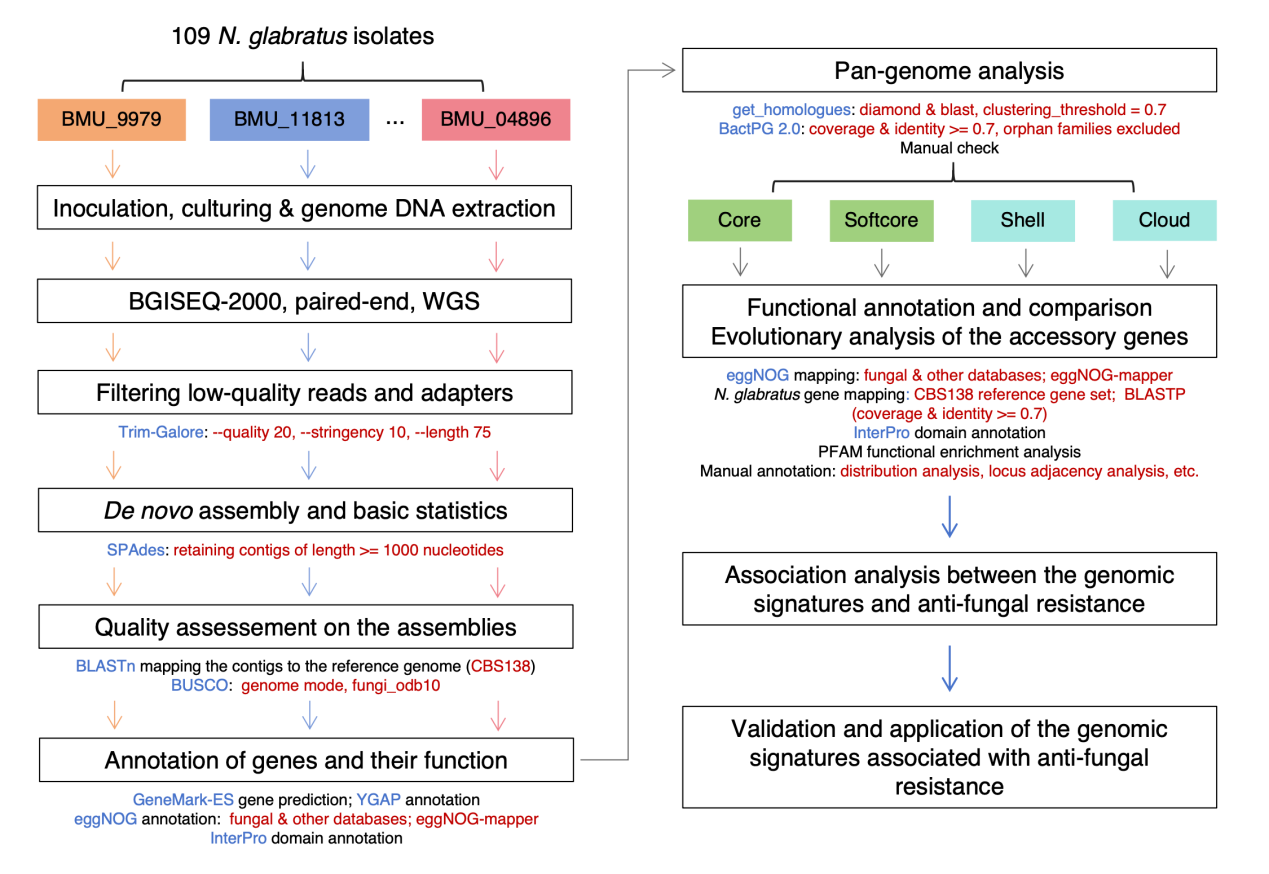


**Figure S1**

**Supplemental Dataset S2. Statistics of the genome assemblies for the 109 *N. glabratus* isolates.**

The genomes of 109 *N. glabratus* isolates were sequenced, filtered for low-quality reads and adapters, and assembled with SPAdes [1]. The number and length distribution of contigs, the total size of the assemblies, and the number of predicted and annotated genes were annotated in Dataset S2 for each isolate. Generally, the sequencing quality is high. The contig number, contig length distribution and the total size of assemblies appeared stable among the isolates. The total length of the assemblies is generally very close to that of the reference genome of *N. glabratus* strain CBS138, suggesting broad coverage and high quality of the genome sequencing and assemblies.

**Supplemental Dataset S3. Quality assessment for the assemblies of 109 *N. glabratus* isolates with BUSCO analysis.**

BUSCO analysis was performed to demonstrate the high quality of the assemblies [2]. The completeness of the genome assemblies and contamination levels could be assessed by the missing rates and duplicated rates with BUSCO analysis, both of which were very low, further suggesting the high quality of the *N. glabratus* assemblies.

**Supplemental Figure S2. Overall coverage of the *N. glabratus* genome assemblies to the genome of reference strain CBS138.**

Contigs of the 109 *N. glabratus* isolates were mapped to the reference genome of N. glabratus (strain CBS138, accession: NC_005967.2). For each reference chromosome, coverages were calculated for each position only if it was aligned by a contig. The depth for each position of the reference chromosomes was colored with a scale preset according to the expected range of contig coverage. Nearly the entire reference genome showed uniform and high-depth coverage by the aligned contigs, demonstrating the ideal coverage and high quality of the *N. glabratus* genome assemblies.


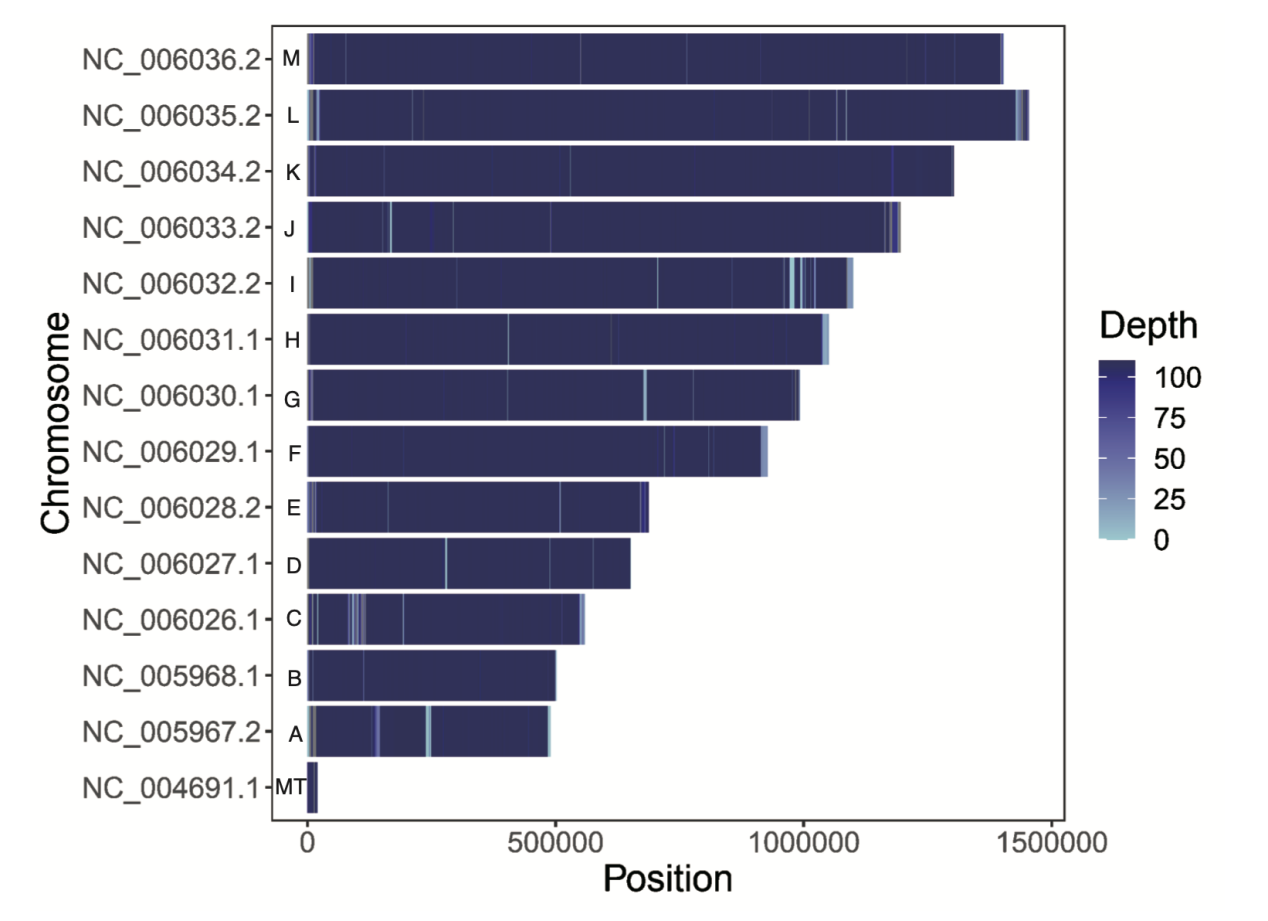
 **Figure S2**

**Supplemental Dataset S4. Per-strain coverage of the *N. glabratus* genome assemblies to the CBS138 genome.**

The genome assemblies of each *N. glabratus* isolate were mapped to the CBS138 genome, and the total aligned length, mapping rate and coverage rate (the percentage of CBS138-covered length to CBS138 genome length) were calculated. The GC content for each genome was also calculated and recorded in Dataset S4 to identify any potential outliers. The reference-coverage rates are generally high, and there is no outlier for GC content, further confirming the high quality of the genome assemblies.

**Supplemental Dataset S5. Per-isolate summary of the *N. glabratus* genome encoding proteins mapped to the proteins encoded by CBS138.**

Protein-encoding genes were predicted with GeneMarker from the genome of each *N. glabratus* isolate [3]. The number of genes was also documented for each isolate in Dataset S2. Mutual alignment was performed between the proteins encoded by each isolate and those encoded by CBS138. The protein mapping rates were calculated for each isolate and documented in Dataset S5. Generally, the number of genes predicted from the genome of each isolate is similar to each other, and the protein-mapping rate to the reference genome is high.

**Supplemental Dataset S6. Per-isolate summary of the annotated *N. glabratus* proteins mapped to the annotated proteins in CBS138.**

Mutual alignment was performed between the eggNOG-annotated proteins for each *N. glabratus* isolate and those of CBS138 [4]. Mapping rates of the annotated proteins were calculated for each isolate and documented in Dataset S6. The number of annotated proteins was also documented for each isolate in Dataset S2. Similar to the number of proteins and protein-mapping rates, the number of annotated genes for each isolate is similar to each other, and the mapping rate of annotated proteins to the reference genome is also high.

Supplemental Datasets 2 ~ 6 and Supplemental Figure S2 together demonstrated the high quality of the sequenced, assembled and annotated genomes for the 109 *N. glabratus* isolates.

**Supplemental Dataset S7. Distribution of the pan-genome families among the 109 *N. glabratus* isolates.**

The pan-genome was analyzed for the 109 *N. glabratus* genomes with both Get_homologues [5] and BactPG2.0 (http://61.160.194.165:3080/ESG/tools/). To reduce the risk of genomic contamination, only a protein family present in more than one isolate was recorded. Both saturation analysis and distribution analysis on pan-genome families were also performed. In total, 6770 reliable pan-genome protein families were identified from the *N. glabratus* genomes. The distribution of these pan-genome families among the 109 isolates was shown in Dataset S7. Saturation analysis indicated that the pan-genome families identified from the 109 genomes are nearly saturated. According to the distribution percentage among the isolates, the families were classified into 4603 core, 200 softcore, 1152 shell and 815 cloud gene families. The shell and cloud genes together constituted the accessory genome. Functional enrichment analysis on the accessory genome encoding protein families demonstrated a large proportion of GLEYA-containing proteins and hyphally regulated cell wall proteins.

**Supplemental Figure S3. Distribution of accessory genes among the 109 *N. glabratus* isolates.**

Most of the isolates showed large but similar number of accessory genes. The distribution of accessory genes, non-fungal originated accessory genes, accessory genes encoded with GLEYA domains was all shown in Figure S3.


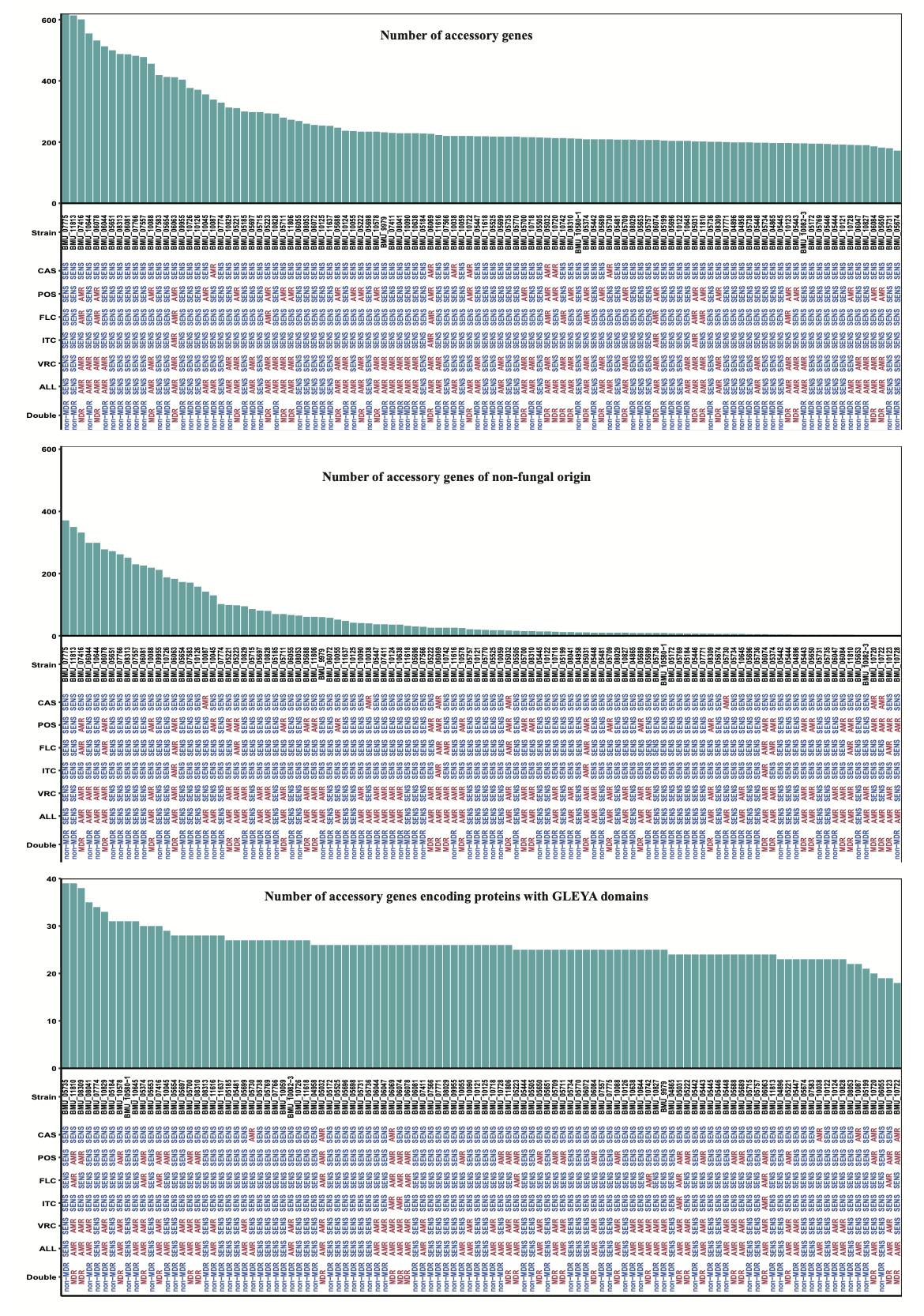


**Figure S3**

**Supplemental Dataset S8. Pan-genome families with copy number variation in the 109 *N. glabratus* genomes.**

Copy number variation (CNV) was analyzed in the *N. glabratus* genomes and summarized in Dataset S8 per pan-genome family and per isolate. In total, there are 274 families showing CNV among the genomes. For each isolate, there are 83-106 families showing with CNV, and 9-19 families with multi-round CNV. The extensive CNV of gene families in *N. glabratus* is consistent with previous studies [6].

**Supplemental Figure S4. Functional enrichment analysis on the *N. glabratus* genes with CNV.**

Functional enrichment was also performed to the protein families duplicated and multi-roundly duplicated in the genomes of *N. glabratus* isolates. GLEYA-containing proteins and hyphally regulated cell wall proteins were also most significantly enriched, consistent with previous observations [6].


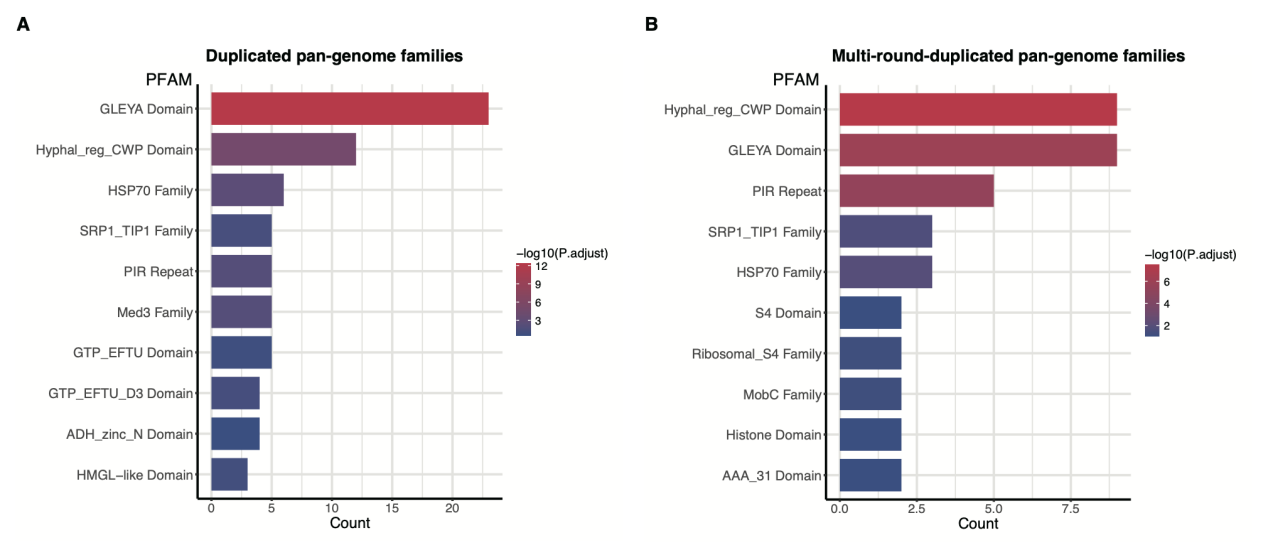


**Figure S4**

**Supplemental Dataset S9. Annotation of a large-fragmental tandom repeat involving 16 gene families in *N. glabratus* isolate BMU05374.**

In BMU05374, a large-fragment duplication was identified in Chromosome A. There are two tandem repeats, each containing 16 genes. It is also an example to show the wide presence of CNV in *N. glabratus*.

**Supplemental Dataset S10. Annotation of non-fungal origin accessory genes in the *N. glabratus* isolates**

There are 6959 genes in the *N. glabratus* isolates showing non-fungus origins, belonging to 1117 pan-genome families. Dataset S10 curated per gene for the isolate, gene accession, pan-genome family, most homologous hit, CBS138 homolog, and the annotation for the alignment and homologous hits. All the non-fungus originated gene families were detected at least in two different *N. glabratus* isolates and more than 64.1% of the families were distributed in three or more isolates, demonstrating the low possibility of genomic contamination.

**Supplemental Dataset S11. Contig continuity analysis for the non-fungal origin accessory genes in the *N. glabratus* isolates**

To further demonstrate that the large percentage of non-fungal origin accessory genes were not caused by genome contamination, the continuity of contigs where the genes are located was analyzed. An underlying hypothesis is that, the contaminant DNA sequences could not be assembled with the genome sequences of the target fungal isolate, and therefore the contigs should be short. Dataset S11 annotated the contig continuity for each non-fungal origin accessory gene in each *N. glabratus* isolate. A summary was also given for length distribution of the involved contigs. The long continuity of the contigs further demonstrated that the non-fungal origin accessory genes in the *N. glabratus* isolates were not caused by contamination.

There are more fungal-origin accessory genes. The non-fungal origin accessory genes are different from fungal-origin accessory genes. *N. glabratus* isolates showed varied number of non-fungal origin accessory genes (Fig. S3), but the number of fungal-origin accessory genes appeared pretty stable. The two types of accessory genes also showed different functional enrichment. The non-fungal origin accessory genes are enriched with those encoding phage integrases and glycosyltransferases, while the fungal-origin accessory genes are enriched with those encoding GLEYA-containing proteins and hyphally regulated cell wall proteins.

In summary, Supplemental Datasets 7 ~ 11 and Supplemental Figures S3 ~ S4 annotated the pan-genome distribution in the *N. glabratus* isolates and the potential origins of accessory genes: duplication and horizontal transfer.

Supplemental Dataset 7, Supplemental Figure S3 and their associated functional annotation or enrichment analysis demonstrated the large plasticity of the *N. glabratus* genome, with a large proportion of accessory genes.

Supplemental Datasets 8 ~ 9 and Supplemental Figure S4 demonstrated the potential contribution of gene duplication in the large accessory genome of *N. glabratus* isolates.

Supplemental Datasets 10 ~ 11 and related analysis demonstrated the potential contribution of horizontal gene transfer from bacteria and other fungi in the large accessory genome of *N. glabratus* isolates.

**Supplemental Dataset S12. Annotation of the GWAS-identified loci for which genetic polymorphisms are significantly associated with antifungal resistance in *N. glabratus*.**

PCA analysis with core-genome SNPs demonstrated that the antifungal resistant and susceptible isolates showed atypical difference, suggesting the existence of possible association between resistant phenotype and genetic variations. Therefore, GWAS analysis was performed on the *N. glabratus* genomes with PLINK [7], to identify the antifungal resistance associated mutations. In total, 1360 loci were identified as being associated with resistance to various antifungals. The most significant resistance-related signatures are located in protein-coding genes or intergenic regions, including *PDR1* associated with FLC- and ITC-resistance, a highly variable intergenic region (HVIGR1) in chromosome C associated with FLC- and POS-resistance, cell wall protein genes such as *AWP2* (CAGL0K00110g) associated with FLC- and POS-resistance, and notably numerous genes encoding proteins with GLEYA domains such as *EPA10* (CAGL0A01284g) associated with FLC-resistance and *EPA22* (CAGL0K00170g) with POS-resistance.

**Supplemental Figure S5. The flowchart of MLFS.**

Comparative genomic analysis gave a large number of signatures associated with antifungal resistance of *N. glabratus*. Therefore, we proposed a Machine Learning based Feature Selection (MLFS) strategy, to reduce the redundant and lowly relevant signatures, select the most relevant signatures, and train models predicting antifungal resistance accurately.


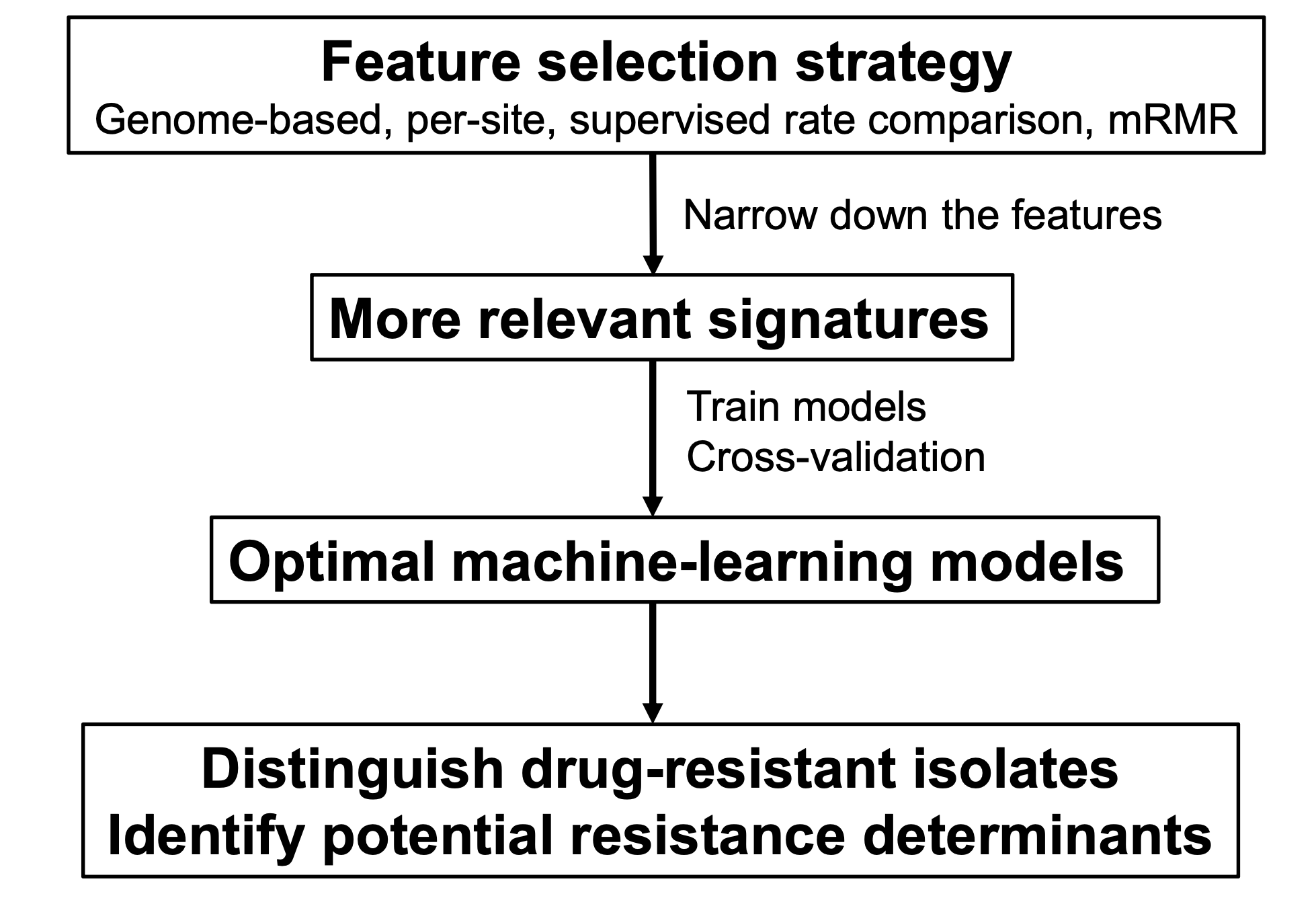


**Figure S5**

**Supplemental Dataset S13. Pan-genome antifungal resistance signatures identified by MLFS.**

The pan-genome genes that could be associated with antifungal resistance were much reduced by MLFS. Many of the identified pan-genome signatures are also consistent with the GWAS results. For example, there are genes encoding hyphally regulated cell wall proteins or GLEYA-containing proteins. Some signatures were reported to be associated with fungal resistance, e.g., an FLC-resistant signature gene *RTT109*, the homolog of which encodes histone acetyltransferase and regulates the expression of the resistance-related *ERG11* gene in *C. albicans* [8].

**Supplemental Dataset S14. Performance of pan-genome feature-based models predicting antifungal resistance of *N. glabratus*.**

Twelve machine-learning models were trained for each antifungal, with 30 most optimized pan-genome features selected with MLFS. The model performance was evaluated based on a 5-fold cross-validation strategy. The average performance was recorded in Dataset S14 for each model. The models were trained for prediction of isolates with azole resistance, since there were only a limited number of isolates showing resistance to echinocandins in this study and the corresponding models could not be established. Generally, the models based on pan-genome signatures showed superior performance in prediction of antifungal, especially azole resistance, in *N. glabratus*.

**Supplemental Dataset S15. Core-genome antifungal resistance signatures identified by MLFS.**

The core-genome signatures that could be associated with antifungal resistance were also narrowed down by MLFS. Several signatures are located in resistance-mediating genes, including *PDR1*, *FPS1* encoding glycerol transporter which is involved in flucytosine- and echinocandin-susceptibility [9], and *AHC1* encoding histone acetyltransferase which influences triazole-susceptibility [10].

**Supplemental Dataset S16. Performance of core-genome feature-based models predicting antifungal resistance of *N. glabratus*.**

Twelve machine-learning models were also trained for each antifungal, with 30 most optimized core-genome signatures selected with MLFS. The model performance was evaluated based on a 5-fold cross-validation strategy. The average performance was recorded in Dataset S16 for each model. Similarly, the models were trained for prediction of isolates with azole resistance, and the models generally showed superior prediction performance.

**Supplemental Dataset S17. Ortholog distribution of typical azole resistance associated genes among *N. glabratus* isolates**

The SNPs of *ERG11*, *SNQ2*, *CDR1* and *CDR2* have been reported to be associated with azole resistance. The ortholog distribution of these genes in the 109 *N. glabratus* are annotated in Dataset S17. The SNPs within these genes are annotated, with which machine learning models were trained to predict antifungal resistance.

**Supplemental Figure S6. Performance comparison between the MLFS models and models based on typical resistance-associated genes.**

The SNPs within *ERG11*, *SNQ2*, *CDR1* and *CDR2* were collected, based on which, LR models were trained. 5-fold cross-validation assessment was performed to these models. The average 5-fold cross-validated ROC curves showed that the models based on these gene polymorphisms as features could predict the AMR, MDR, FLC and POS with an average AUC of 0.54~0.66, much poorer than those core-genome or pan-genome models based on MLFS screened signatures.


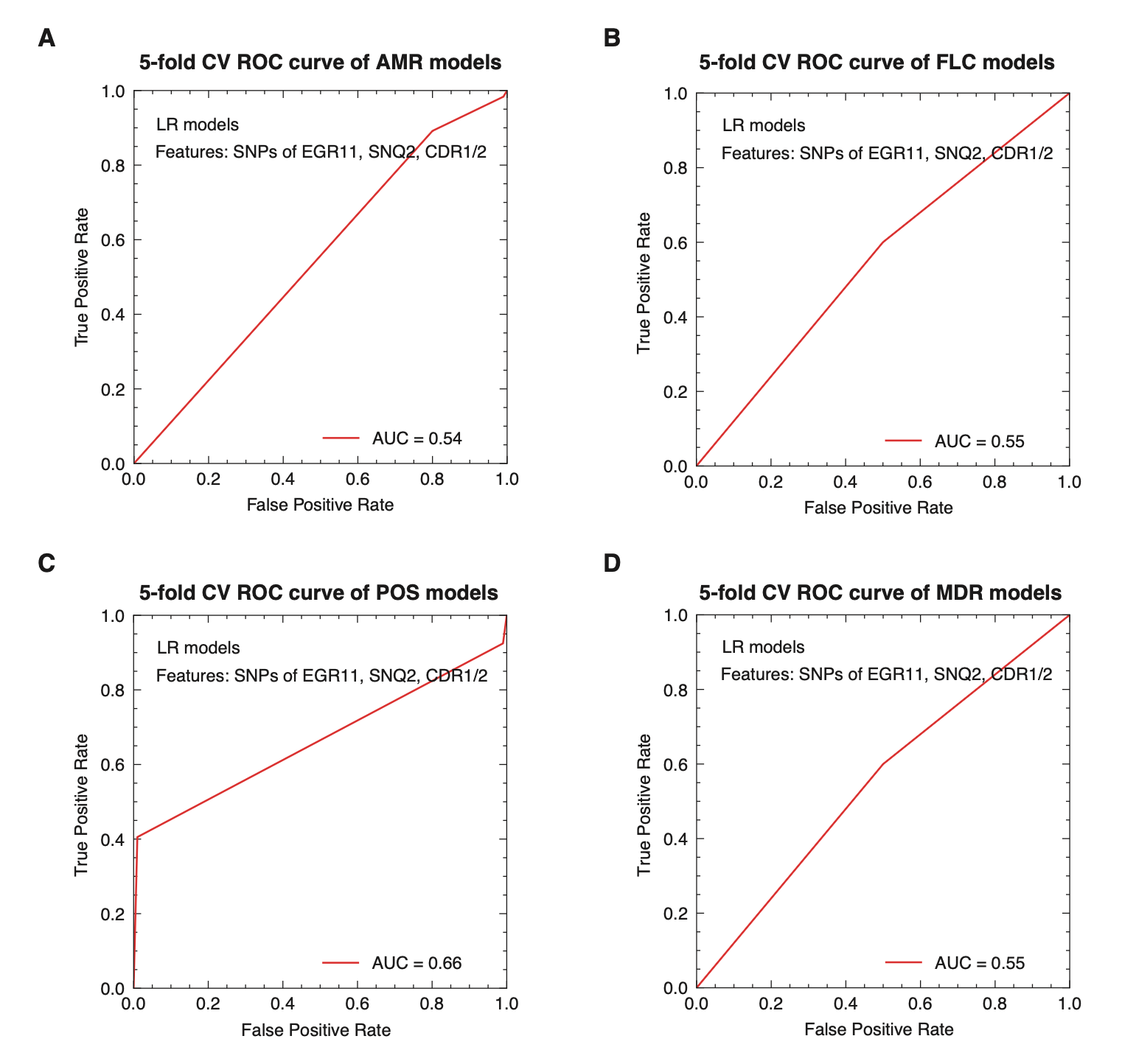


**Figure S6**

**Supplemental Figure S7. The 5-fold cross-validated ROC curves of models based on extensive pan-genome (ePG) features.**

We also tested other types of genomic signatures such as CNV. We integrated CNV features with original pan-genome features, together called ePG features. The ePG features were filtered with a similar MLFS strategy. With the 30 top optimized ePG features, LR models were trained. Comparison was performed between these ePG models and PG models. The average 5-fold cross-validated ROC curves were shown in Fig. S7. The results demonstrated that inclusion of CNV features did not significantly improve the prediction performance of the machine-learning models.


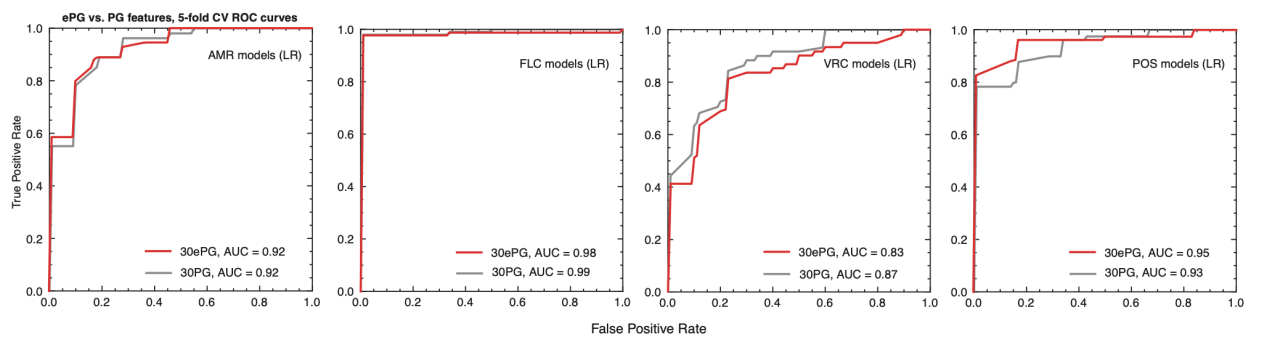


**Figure S7**

**Supplemental Dataset S18. The optimized parameters for the models**.

The optimized parameters were annotated for each pan-genome or core-genome model based on the best 10, 20 or 30 features selected by MLFS.

In summary, Supplemental Datasets 12 ~ 18 and Supplemental Figures 5 ~ 7 showed the genetic association of azole resistance in *N. glabratus,* and the effectiveness of MLFS in facilitating identification of genetic determinants for antifungal resistance and accurate prediction of *N. glabratus* isolates with the phenotype of antifungal resistance.

**Supplemental Dataset S19. Differential pan-genome families identified from the comparative genomic analysis between echinocandin-resistant and -susceptible isolates**.

There were very limited number of *N. glabratus* isolates in this study showing resistance to echinocandins (Dataset S1). Therefore, the MLFS strategy was not suitable for the analysis of echinocandin-associated signatures. However, three isolates, BMU_10720, BMU_10722 and BMU_11616, were identified, which were closely clustered in the phylogenetic tree, but with the former two being resistant and the latter one susceptible to echinocandins (Dataset S1). Comprehensive comparative genomic analysis was performed between the isolates with the two different phenotype, including distribution of pan-genome families, CNV, and genetic polymorphisms that affect amino acids. The consensus differential genes were identified and annotated in Dataset S19.

**Supplemental Figure S8. Functional enrichment analysis on the different protein families between echinocandin- resistant and susceptible isolates.**

Between phylogetically close *N. glabratus* isolates BMU_10720/BMU_10722 and BMU_11616, which are resistant and susceptible to echinocandins, except for the genes with consensus SNPs and INDELs that could alter the amino acids (Dataset S19), other types of differential gene families showed significant functional enrichment. GLEYA domain containing proteins or cell wall proteins were frequently enriched.


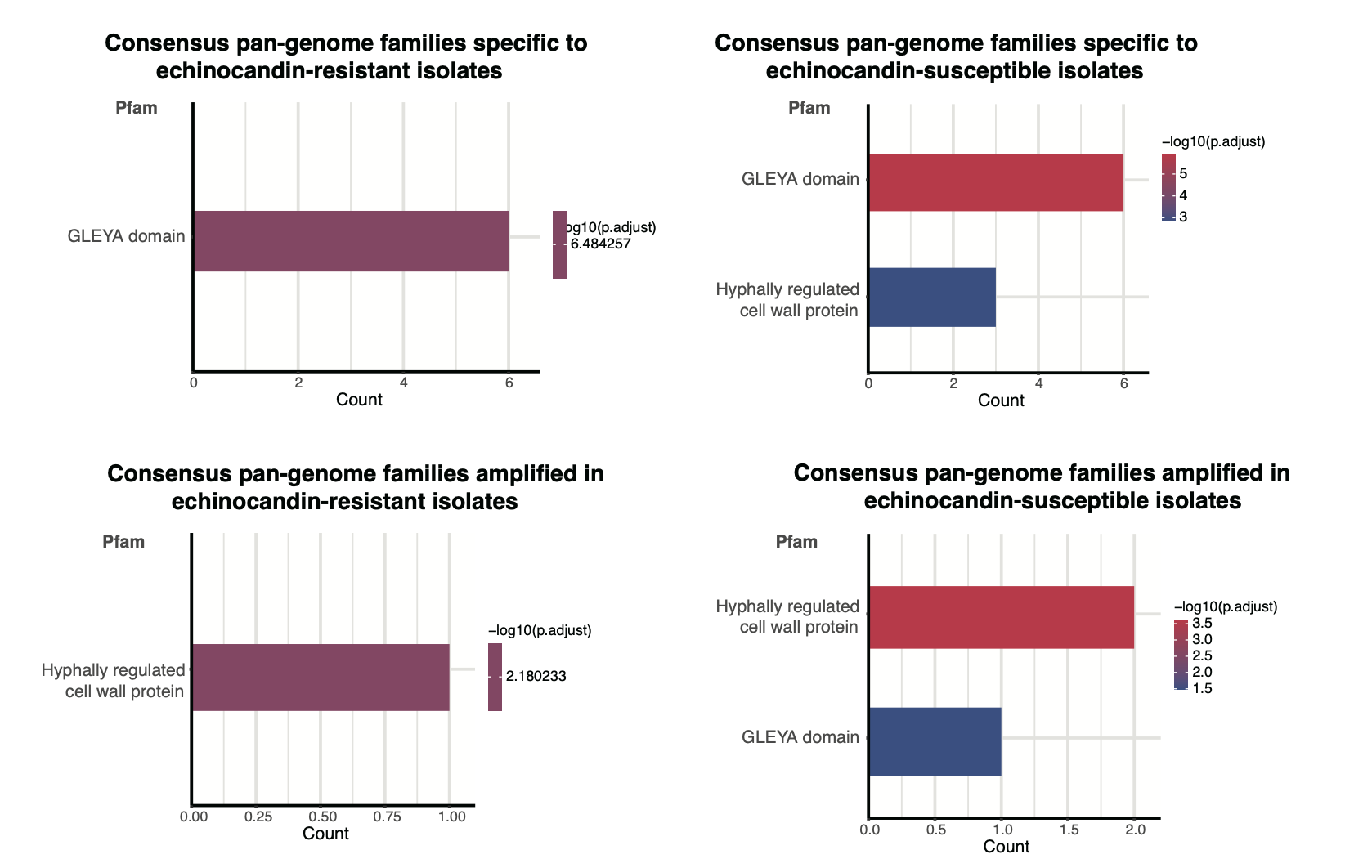


**Figure S8**

In summary, by comparing the genomes of phylogenetically close isolates with different resistant phenotypes, interesting clues could be obtained about the genetic differences possibly related with echinocandin resistance. However, besides echinocandin, the three *N. glabratus* isolates under investigation also show different resistance to other antifungals, such as POS and VRC. Therefore, the genetic variations identified by comparative genomic analysis could be biased to explain the echinocandin resistance. More isolates with strict stratification and corrections for resistance to other drugs are required to make more reliable observations for the mechanisms of echinocandin resistance.

Finally, we also selected two novel core-genome signatures, i.e., I380L and Y682C in *PDR1*, performed wet-lab experiments, and confirmed their roles in conferring triazole-resistance in *N. glabratus*.

In conclusion, this study provides a comprehensive view of plastic genome of *N. glabratus* comprising an expanded accessory genome. The expanded accessory genome could be due to the frequent gene duplication and fungal or non-fungal gene transfer. In the study, we also proposed an MLFS strategy, which could assist identification of resistance determinants and training accurate models predicting antifungal resistance, together with traditional comparative genomic analysis. Two novel signatures identified by MLFS, Y682C and I380L mutations in *PDR1*, were further confirmed experimentally, conferring triazole resistance in *N. glabratus*.

**References**

1. Prjibelski A, Antipov D, Meleshko D, et al. Using SPAdes de novo assembler. Curr Protoc Bioinformatics. 2020;70(1):e102. doi: 10.1002/cpbi.102.
2. Simão FA, Waterhouse RM, Ioannidis P, et al. BUSCO: assessing genome assembly and annotation completeness with single-copy orthologs. Bioinformatics. 2015;31(19):3210-2. doi: 10.1093/bioinformatics/btv351.
3. Ter-Hovhannisyan V, Lomsadze A, Chernoff YO, et al. Gene prediction in novel fungal genomes using an ab initio algorithm with unsupervised training. Genome Res. 2008;18(12):1979-90. doi: 10.1101/gr.081612.108.
4. Cantalapiedra CP, Hernández-Plaza A, Letunic I, et al. eggNOG-mapper v2: functional annotation, orthology assignments, and domain prediction at the metagenomic scale. Mol Biol Evol. 2021;38(12):5825-9. doi: 10.1093/molbev/msab293.
5. Contreras-Moreira B, Vinuesa P. GET_HOMOLOGUES, a versatile software package for scalable and robust microbial pangenome analysis. Appl Environ Microbiol. 2013;79(24):7696-701.  doi: [10.1128/AEM.02411-13](https://doi.org/10.1128/AEM.02411-13)
6. Carreté L, Ksiezopolska E, Pegueroles C, et al. Patterns of genomic variation in the opportunistic pathogen *Candida glabrata* suggest the existence of mating and a secondary association with humans. Curr biol. 2018;28(1):15-27.e7. doi: 10.1016/j.cub.2017.11.027.
7. Purcell S, Neale B, Todd-Brown K, et al. PLINK: a tool set for whole-genome association and population-based linkage analyses. Am J Hum Genet. 2007;81(3):559-75. doi: 10.1086/519795.
8. Jain P, Garai P, Sethi SC, et al. Modulation of azole sensitivity and filamentation by GPI15, encoding a subunit of the first GPI biosynthetic enzyme, in *Candida albicans*. Sci Rep. 2019;9(1):8508. doi: 10.1038/s41598-019-44919-4.
9. Beese-Sims SE, Pan SJ, Lee J, et al. Mutants in the *Candida glabrata* glycerol channels are sensitized to cell wall stress. Eukaryot Cell. 2012;11(12):1512-9. doi: 10.1128/ec.00231-12.
10. O’Kane C, O’Connell M, Hyland E. Probing the role of histone modifications in the evolution of pathogenicity in *Candida glabrata*. Access Microbiology. 2019;1(1A). doi: 10.1099/acmi.ac2019.po0288.
