# Supplementary figures and images for: Genome analysis and machine learning-based feature selection strategy reveal potential drug-resistance determinants in Nakaseomyces glabratus
Source: Emerg Microbes Infect. 2025 Dec 13;14(1):2595789. doi: 10.1080/22221751.2025.2595789 (PMC12704144; doi:10.1080/22221751.2025.2595789)

ePG vs. PG features, 5-fold CV ROC curves

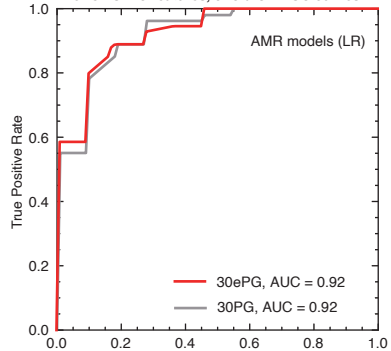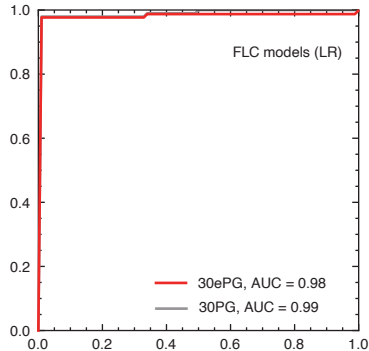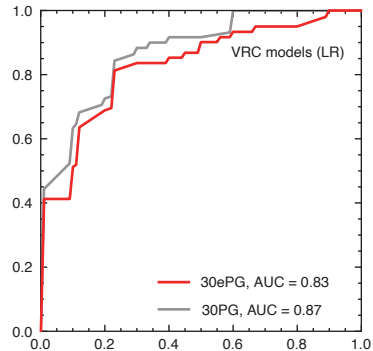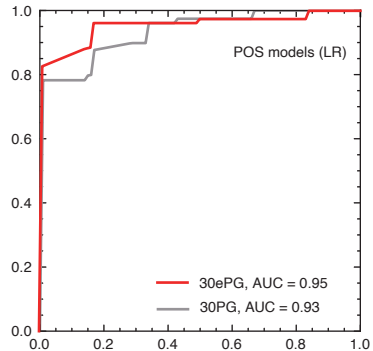

False Positive Rate

Supplement: Fig_S7.pdf [file TEMI_A_2595789_SM5788.pdf]

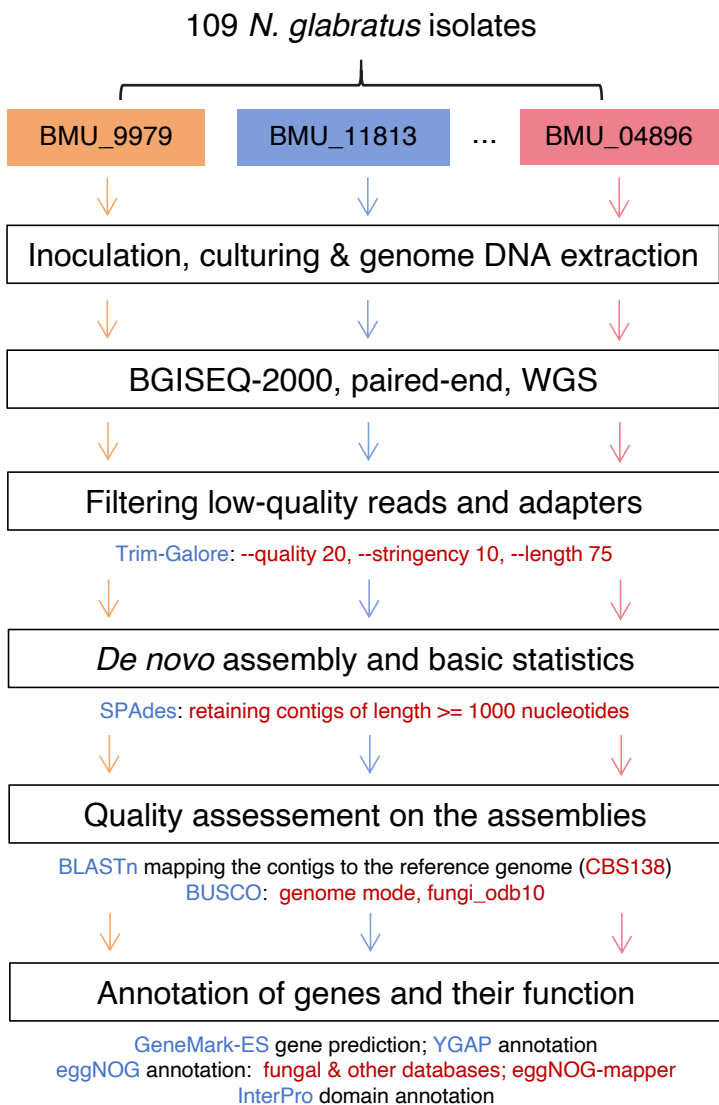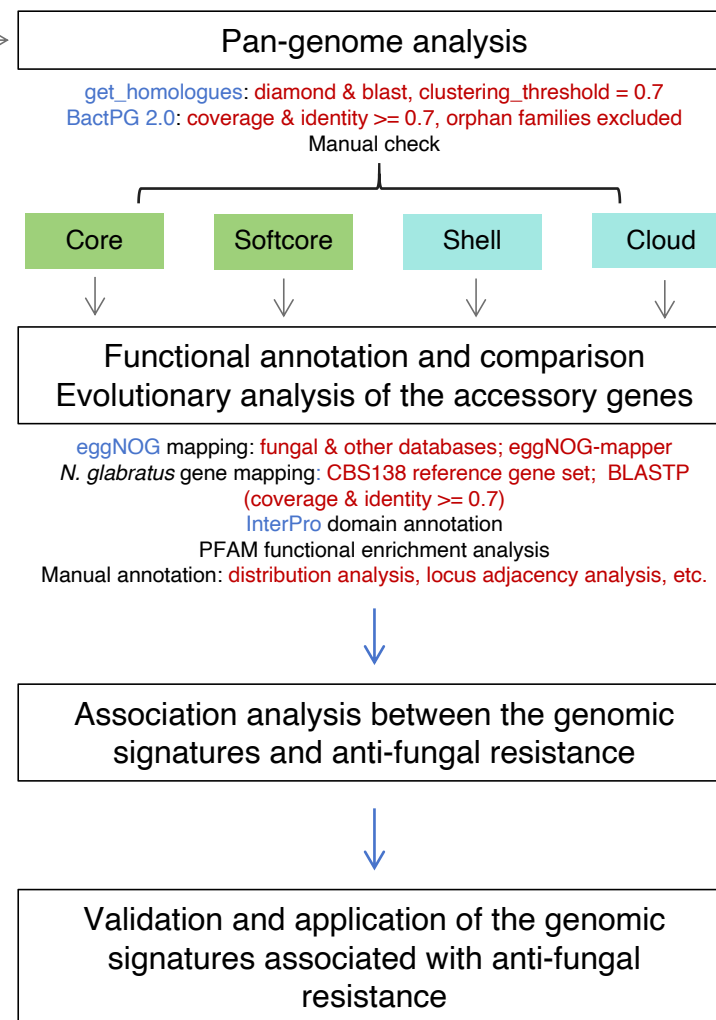

Supplement: Fig_S1_Pipeline.pdf [file TEMI_A_2595789_SM5787.pdf]

**A**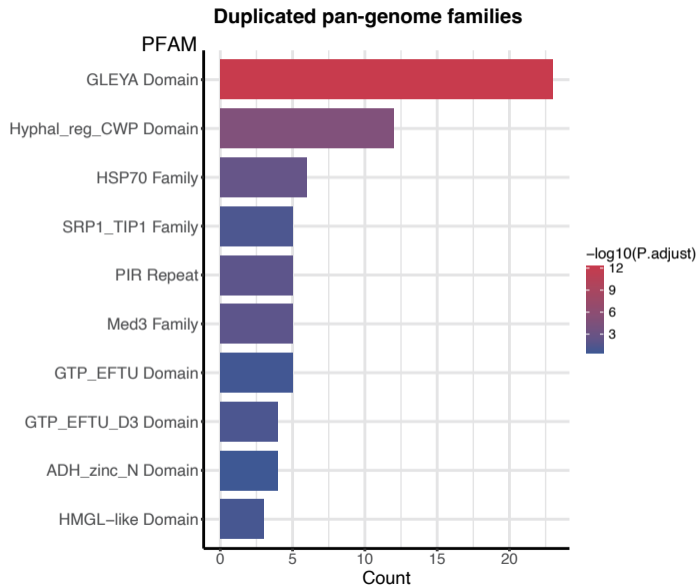**B**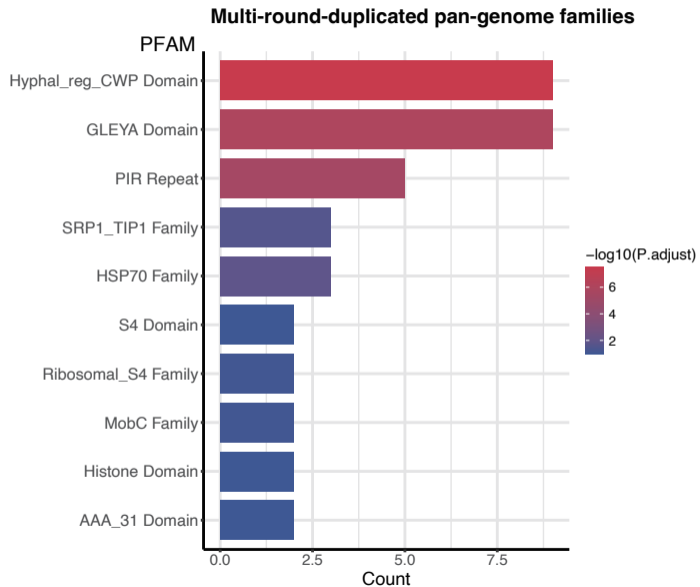

Supplement: Fig_S4.pdf [file TEMI_A_2595789_SM5786.pdf]

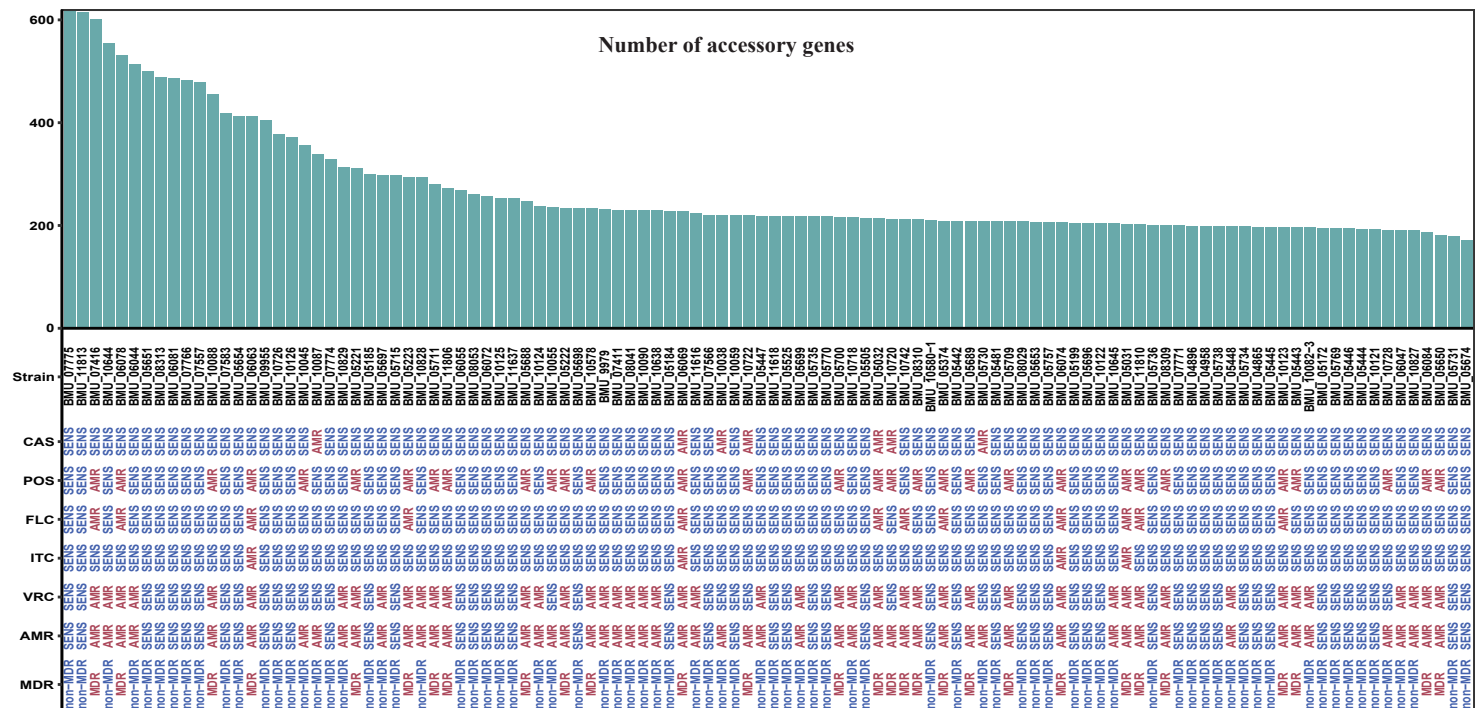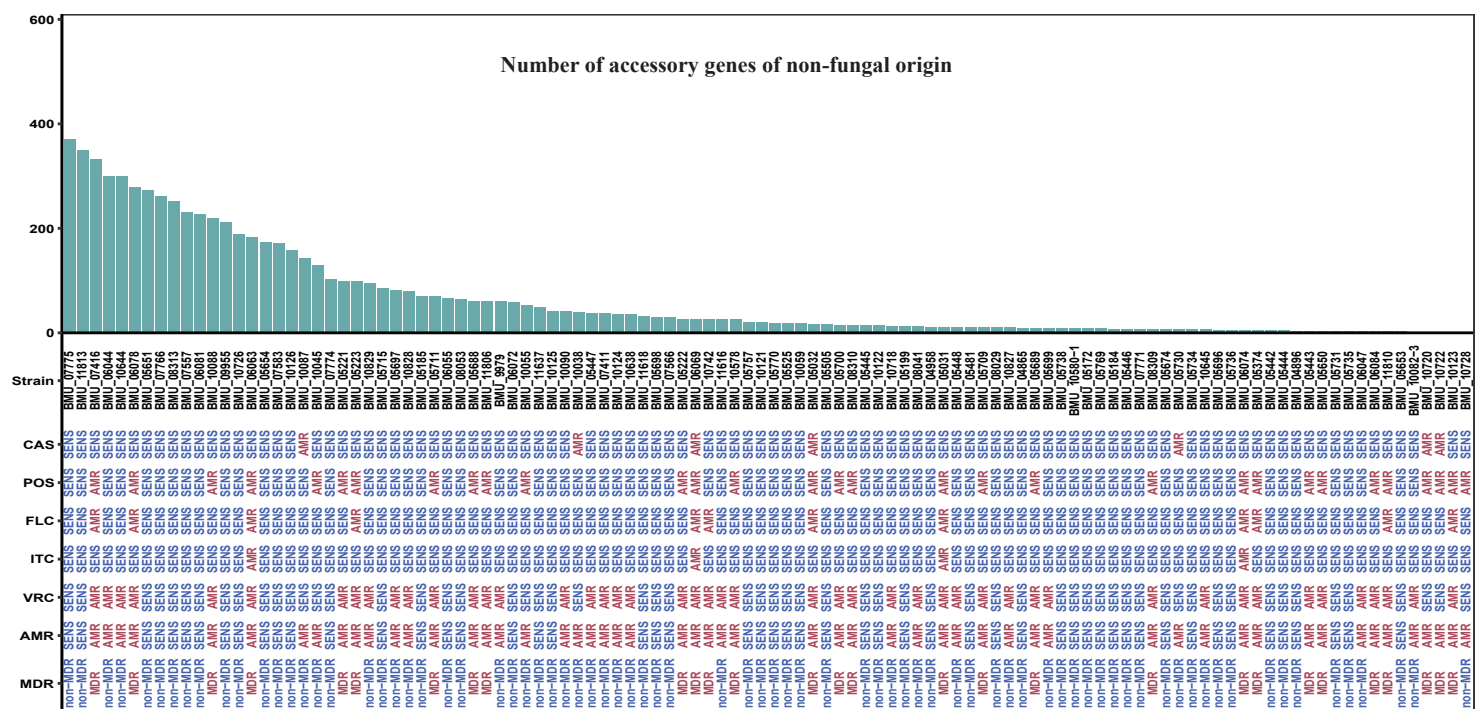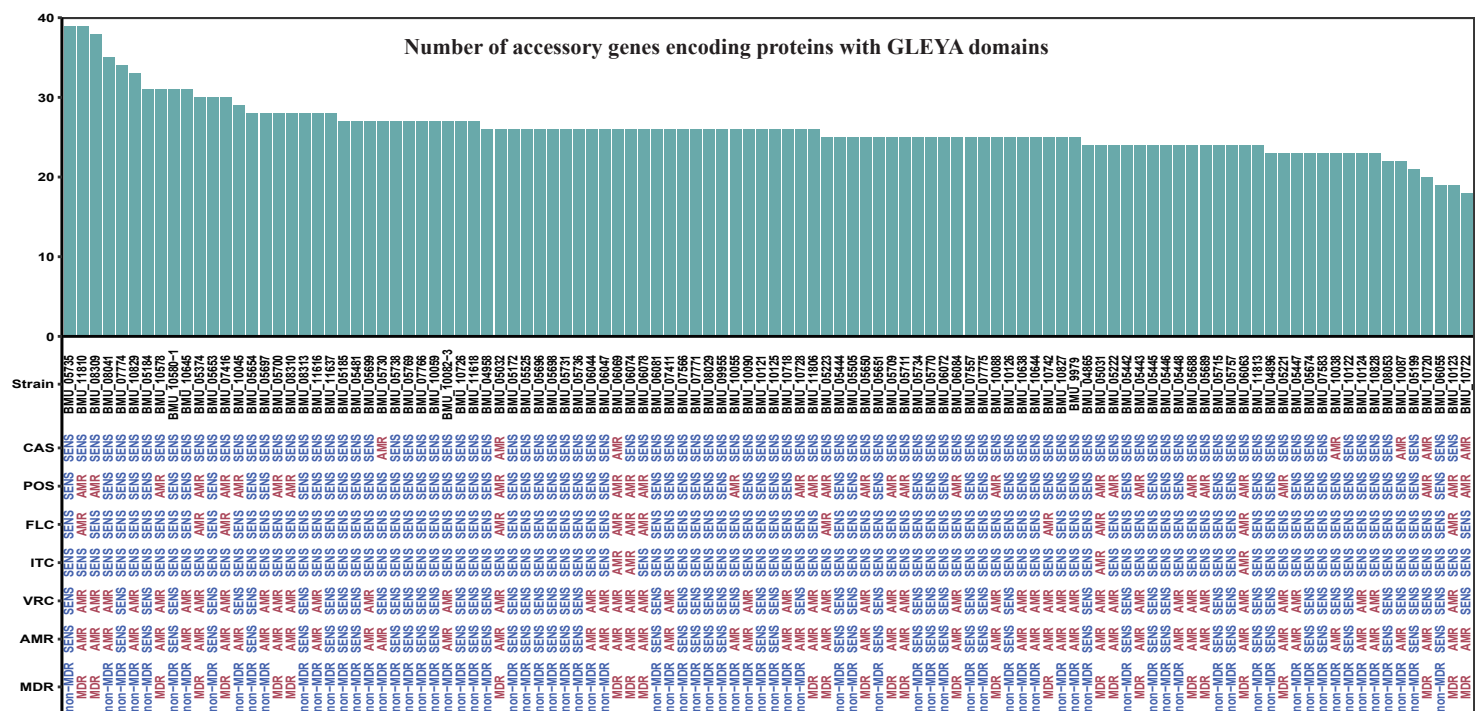

Supplement: Fig_S3_revised.pdf [file TEMI_A_2595789_SM5783.pdf]

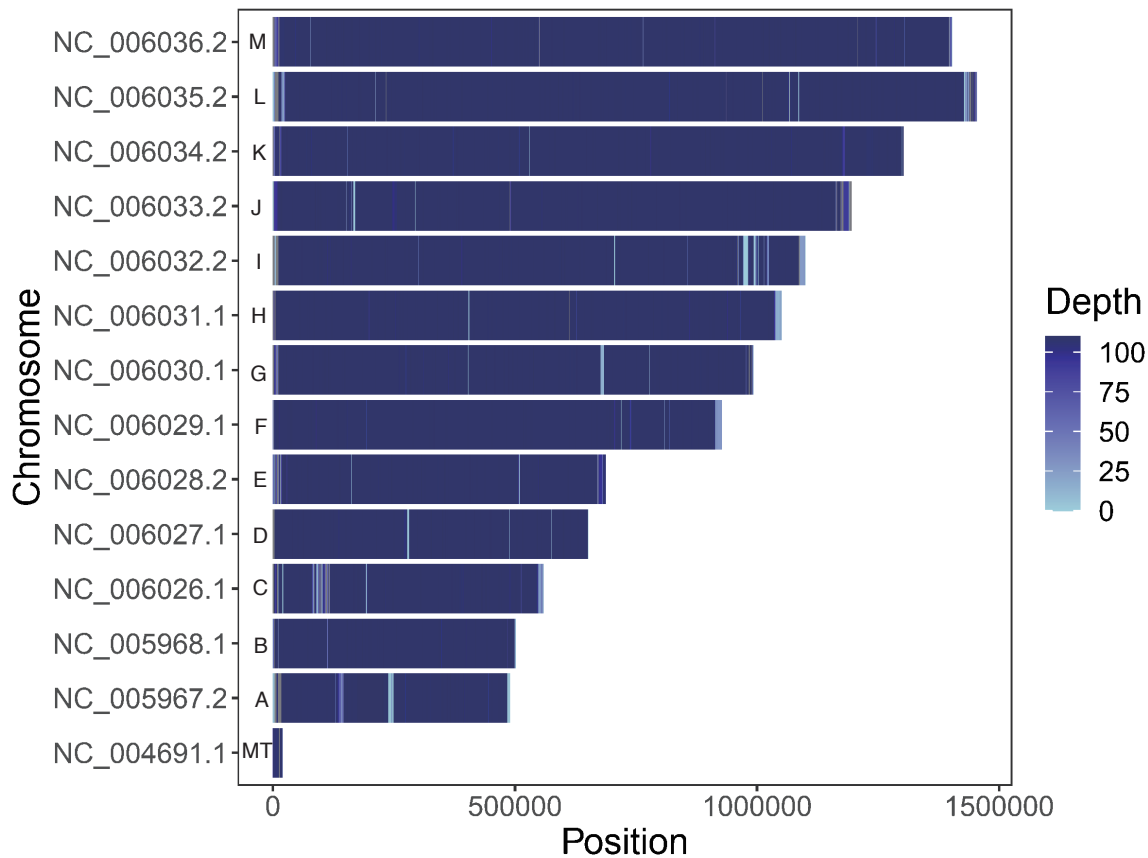

Supplement: Fig_S2.pdf [file TEMI_A_2595789_SM5781.pdf]
