# Supplementary material for: Genome analysis and machine learning-based feature selection strategy reveal potential drug-resistance determinants in Nakaseomyces glabratus
Source: Emerg Microbes Infect. 2025 Dec 13;14(1):2595789. doi: 10.1080/22221751.2025.2595789 (PMC12704144; doi:10.1080/22221751.2025.2595789)
Supplement: Fig_S8.pdf [file TEMI_A_2595789_SM5785.pdf]

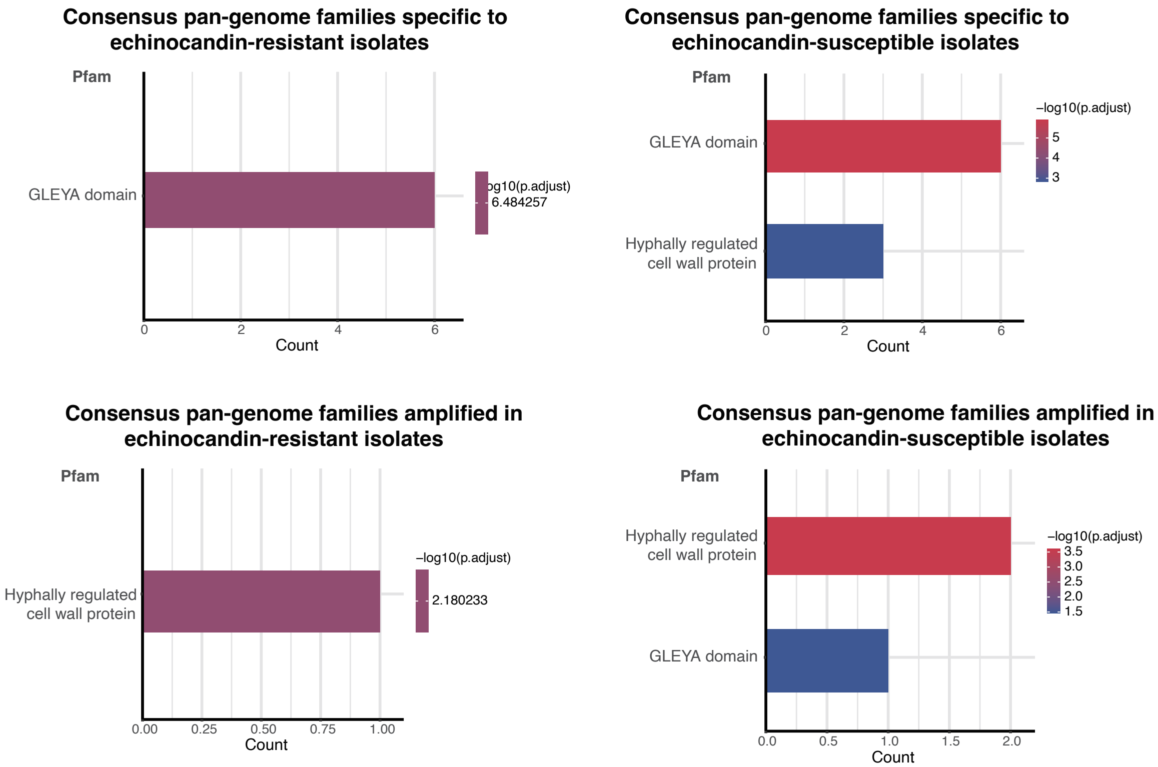

Consensus pan-genome families specific to echinocandin-resistant isolates
Pfam
GLEYA domain
0
2
4
6
Count
og10(p.adjust)
6.484257
Consensus pan-genome families specific to echinocandin-susceptible isolates
Pfam
GLEYA domain
Hyphally regulated cell wall protein
0
2
4
6
Count
−log10(p.adjust)
5
4
3
Consensus pan-genome families amplified in echinocandin-resistant isolates
Pfam
Hyphally regulated cell wall protein
0.00
0.25
0.50
0.75
1.00
Count
−log10(p.adjust)
2.180233
Consensus pan-genome families amplified in echinocandin-susceptible isolates
Pfam
Hyphally regulated cell wall protein
GLEYA domain
0.0
0.5
1.0
1.5
2.0
Count
−log10(p.adjust)
3.5
3.0
2.5
2.0
1.5
