# Supplementary material for: Genome analysis and machine learning-based feature selection strategy reveal potential drug-resistance determinants in Nakaseomyces glabratus
Source: Emerg Microbes Infect. 2025 Dec 13;14(1):2595789. doi: 10.1080/22221751.2025.2595789 (PMC12704144; doi:10.1080/22221751.2025.2595789)
Supplement: Fig_S6.pdf [file TEMI_A_2595789_SM5776.pdf]

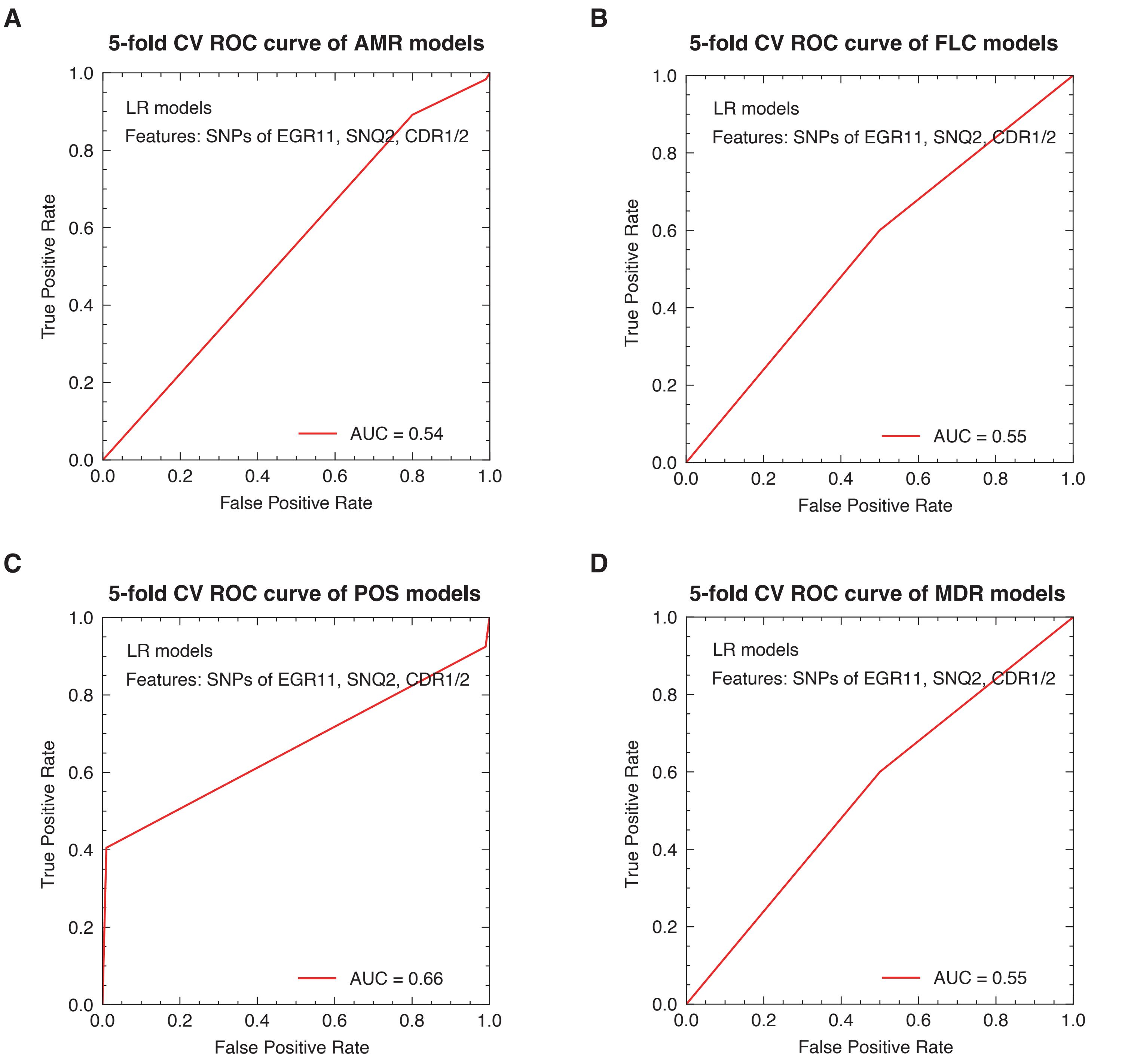
A
5-fold CV ROC curve of AMR models
LR models
Features: SNPs of EGR11, SNQ2, CDR1/2
AUC = 0.54
True Positive Rate
False Positive Rate
0.0
0.2
0.4
0.6
0.8
1.0
B
5-fold CV ROC curve of FLC models
LR models
Features: SNPs of EGR11, SNQ2, CDR1/2
AUC = 0.55
C
5-fold CV ROC curve of POS models
LR models
Features: SNPs of EGR11, SNQ2, CDR1/2
AUC = 0.66
D
5-fold CV ROC curve of MDR models
LR models
Features: SNPs of EGR11, SNQ2, CDR1/2
AUC = 0.55
