# Supplementary material for: Genome analysis and machine learning-based feature selection strategy reveal potential drug-resistance determinants in Nakaseomyces glabratus
Source: Emerg Microbes Infect. 2025 Dec 13;14(1):2595789. doi: 10.1080/22221751.2025.2595789 (PMC12704144; doi:10.1080/22221751.2025.2595789)
Supplement: Description_on_supplemental_materials.docx [file TEMI_A_2595789_SM5774.docx]

**Supplemental Figures Legends**

**Fig. S1. Workflow for genome analysis in this study.**

**Fig. S2. Coverage of the 109 genomes of *Nakaseomyces glabratus* isolates of the chromosomes in the reference genome.** Each genome was assembled to the chromosome level, with average 99.54% coverage of the 13 chromosomes (A-M) and a mitochondrion (MT) chromosome. Reference genome of *Nakaseomyces glabratus*: GCF_000002545.3.

**Fig. S3. Relationship of the 109 *Nakaseomyces glabratus* isolates between drug resistance and the number of all accessory genes (A), accessory genes of non-fungal origins (B), and accessory genes encoding proteins with GLEYA domains (C).** The number of accessory genes and genes of non-fungal origins as well as the genes encoding GLEYA-containing proteins were various in the 109 isolates, despite that no direct association with antifungal susceptibility in *N. glabratus* was observed.

**Fig. S4. Functional enrichment analysis on the duplicated pan-genome families in the 109 *N. glabratus* isolates.** Significantly enriched PFAMs were shown for all the duplicated pan-genome families **(A)** and the multi-round-duplicated (≥3) pan-genome families **(B)**, respectively.

**Fig. S5. Workflow for machine-learning-based feature selection (MLFS) strategy.**

**Fig. S6. Performance of models based on the SNPs of well-known AMR-related genes for predicting the resistance in 109 *N. glabratus isolates* to different antifungal drugs or combinations.** The AUC of ROC curves and 5-fold cross-validation (CV) was used for the performance evaluation. Logistic Regression (LR) models were trained.

**Fig. S7. The 5-fold cross-validated ROC curves of models based on extensive pan-genome (ePG) features.** The LR models with 30 optimized features were shown. 30ePG, models with 30 ePG features; 30PG, models with 30PG features.

**Fig. S8. Functional enrichment analysis on the different protein families between echinocandin-resistant and -susceptible isolates.**

**Supplemental Datasets**

**Dataset S1** (separate file). Information of 109 *Nakaseomyces glabratus* isolates including sample types, isolation time, antifungal susceptibilities, *PDR1* mutations, and their National Microbiology Data Center (NMDC) numbers. Primers used in this study are listed in the sheet.

**Dataset S2** (separate file). Detailed statistics for the individual assembly of the 109 *N. glabratus* genomes.

**Dataset S3** (separate file). BUSCO analysis for the individual assembly of the 109 *N. glabratus* genomes.

**Dataset S4** (separate file). Statistics for mapping of the 109 *N. glabratus* genome assemblies to the genome of the reference strain CBS138.

**Dataset S5** (separate file). Statistics for mapping of the proteins annotated from the 109 *N. glabratus* genomes to those of the reference strain CBS138.

**Dataset S6** (separate file). Statistics for mapping of the eggNOG-annotated proteins of the 109 *N. glabratus* isolates to the eggNOG-annotated proteins of the reference strain CBS138.

**Dataset S7** (separate file). The pan-genome of 109 *N. glabratus* isolates.

**Dataset S8** (separate file). The pan-genome families of 109 *N. glabratus* isolates with gene copy number variation.

**Dataset S9** (separate file). The large fragmental duplication in *N. glabratus* isolate BMU05374.

**Dataset S10** (separate file). Annotation for the accessory genes of 109 *N. glabratus* isolates with non-fungal origins. The closest homologs of non-fungal origins and in CBS138, as well as the homology levels, were both annotated.

**Dataset S11** (separate file). The contigs where the accessory genes with non-fungal origins and their continuity statistics.

**Dataset S12** (separate file). Annotation of the genetic signatures linked to specific drug resistance identified by genome-wide association analysis.

**Dataset S13** (separate file). Top 30 pan-genome based genetic features used for model training.

**Dataset S14** (separate file). 5-fold cross-validation assessment of the models predicting resistant isolates based on pan-genome features.

**Dataset S15** (separate file). Top 30 core-genome based genetic features used for model training.

**Dataset S16** (separate file). 5-fold cross-validation assessment of the models predicting resistant isolates based on core-genome features.

**Dataset S17** (separate file). Distribution of the genes well-known to be associated with azole resistance in the 109 *N. glabratus* isolates.

**Dataset S18** (separate file)**.** The optimized parameters for the machine learning (ML) models.

**Dataset S19** (separate file). Differential pan-genome families identified from the comparative genomic analysis between echinocandin-resistant and -susceptible isolates.

**Supplemental Table S1** - for reviewers only. The antifungal-resistance prediction performance for the preliminary external dataset.
